# Dimensions of biodiversity loss: Spatial mismatch in land‐use impacts on species, functional and phylogenetic diversity of European bees

**DOI:** 10.1111/ddi.12638

**Published:** 2017-09-13

**Authors:** Adriana De Palma, Michael Kuhlmann, Rob Bugter, Simon Ferrier, Andrew J. Hoskins, Simon G. Potts, Stuart P.M. Roberts, Oliver Schweiger, Andy Purvis

**Affiliations:** ^1^ Department of Life Sciences Natural History Museum London SW7 5BD UK; ^2^ Department of Life Sciences Imperial College London Ascot SL5 7PY UK; ^3^ Zoological Museum University of Kiel Kiel Germany; ^4^ Wageningen Environmental Research (Alterra) Wageningen P.O. Box 47, 6700 AA The Netherlands; ^5^ CSIRO Land and Water Canberra ACT 2601 ACT Australia; ^6^ Centre for Agri‐Environmental Research School of Agriculture, Policy and Development The University of Reading Reading RG6 6AR UK; ^7^ Helmholtz Centre for Environmental Research—UFZ Department of Community Ecology 06120 Halle Germany

**Keywords:** agricultural intensification, land‐use conversion, non‐random species loss, pollinator diversity

## Abstract

**Aim:**

Agricultural intensification and urbanization are important drivers of biodiversity change in Europe. Different aspects of bee community diversity vary in their sensitivity to these pressures, as well as independently influencing ecosystem service provision (pollination). To obtain a more comprehensive understanding of human impacts on bee diversity across Europe, we assess multiple, complementary indices of diversity.

**Location:**

One Thousand four hundred and forty six sites across Europe.

**Methods:**

We collated data on bee occurrence and abundance from the published literature and supplemented them with the PREDICTS database. Using Rao's Quadratic Entropy, we assessed how species, functional and phylogenetic diversity of 1,446 bee communities respond to land‐use characteristics including land‐use class, cropland intensity, human population density and distance to roads. We combined these models with statistically downscaled estimates of land use in 2005 to estimate and map—at a scale of approximately 1 km^2^—the losses in diversity relative to semi‐natural/natural baseline (the predicted diversity of an uninhabited grid square, consisting only of semi‐natural/natural vegetation).

**Results:**

We show that—relative to the predicted local diversity in uninhabited semi‐natural/natural habitat—half of all EU27 countries have lost over 10% of their average local species diversity and two‐thirds of countries have lost over 5% of their average local functional and phylogenetic diversity. All diversity measures were generally lower in pasture and higher‐intensity cropland than in semi‐natural/natural vegetation, but facets of diversity showed less consistent responses to human population density. These differences have led to marked spatial mismatches in losses: losses in phylogenetic diversity were in some areas almost 20 percentage points (pp.) more severe than losses in species diversity, but in other areas losses were almost 40 pp. less severe.

**Main conclusions:**

These results highlight the importance of exploring multiple measures of diversity when prioritizing and evaluating conservation actions, as species‐diverse assemblages may be phylogenetically and functionally impoverished, potentially threatening pollination service provision.

## 1 INTRODUCTION

Bees are widely considered to be the most important group of pollinators, especially in temperate systems (Klein et al., 2007). Although the importance of other insect pollinators has probably been underestimated (Orford, Vaughan, & Memmott, 2015; Rader et al., 2016), declines in bee diversity could have serious consequences for pollination services. At the local scale, bees can be adversely impacted by human‐dominated land uses, such as intensively managed cropland (De Palma et al., 2015; Forrest, Thorp, Kremen, & Williams, 2015), with increased external inputs having both direct impacts (e.g., Woodcock et al., 2016 show that exposure to neonicotinoids is associated with higher rates of bee population extinction) and indirect effects (e.g., use of fertilizers can reduce plant diversity and thus resources Kleijn et al., 2009; Roulston & Goodell, 2011). On the other hand, bee species may benefit from some human impacts, such as the presence of post‐industrial land, such as brownfield sites (Baldock et al., 2015). Pressures in the surrounding landscape can also influence bees, for instance, habitat degradation (Kennedy et al., 2013) and fragmentation (Steffan‐Dewenter, 2003). In Europe, although declines in bee species richness appear to have slowed since 1990 (Carvalheiro et al., 2013), there is still much concern about diversity losses as agricultural intensification, abandonment and urbanization are set to continue (Stoate et al., 2009; Verburg et al., 2006).

No single measure of assemblage diversity can fully capture ecosystem service provision or sensitivity to land‐use change. Species diversity of pollinators can enhance plant reproductive success, increasing crop yield and stability (Albrecht, Schmid, Hautier, & Muller, 2012; Garibaldi et al., 2011; Rogers, Tarpy, & Burrack, 2014), but other aspects of assemblage diversity can also independently influence ecosystem service provision. Functionally, diverse bee communities help maintain plant diversity (Fontaine, Dajoz, Meriguet, & Loreau, 2006), enhance plant reproduction (Albrecht et al., 2012) and increase the volume, quality and stability of crop yields (Garibaldi et al., 2015; Hoehn, Tscharntke, Tylianakis, & Steffan‐Dewenter, 2008). Higher phylogenetic diversity can also enhance ecosystem service provision (e.g., by increasing the stability of ecosystem service provision in plants: Cadotte, Dinnage, & Tilman, 2012), although little evidence is available for its impact on pollination services. As functional traits are often phylogenetically conserved (Freckleton, Harvey, & Pagel, 2002), phylogenetic diversity can relate to the functional diversity of communities (Srivastava, Cadotte, Macdonalda, Marushia, & Mirotchnick, 2012), but the two may not be interchangeable (Flynn, Mirotchnick, Jain, Palmer, & Naeem, 2011); phylogenetic diversity may better represent ecological differences (Srivastava et al., 2012) including plant‐pollinator interactions (Rezende, Lavabre, Guimarães, Jordano, & Bascompte, 2007).

Although these facets of diversity are usually positively correlated with each other (Stevens & Tello, 2014), they may respond differently to pressures. For example, Forrest et al. (2015) showed that relative to natural habitat, low‐intensity cropland could maintain species diversity but not functional diversity. To obtain a more comprehensive understanding of human impacts on biodiversity, it is important therefore to assess multiple, complementary indices of diversity (Vandewalle et al., 2010). To date, little research has explored whether different facets of bee diversity show similar responses to land use and related pressures or whether the losses in diversity are congruent across space.

We use data from 1,446 sites and 317 bee species to provide the first continental assessment of how conceptually matched measures of species, functional and phylogenetic diversity of bee communities vary with land use and related pressures across Europe. European bees provide a suitable focus, as data on their abundances and ecological traits are readily available; data collations for other regions and most other invertebrate taxa are less complete (Hudson et al., 2017). We estimate bee diversity given the land‐use class and management intensity of cropland at the local level and the estimated impacts of human population density (a general proxy for habitat disturbance) and distance to roads (a proxy for fragmentation) in the surrounding area. By combining the resulting models with fine‐resolution maps of these pressures, we estimate the losses of bee diversity for each 1 km^2^ grid square across the EU27 region, relative to the predicted diversity in an uninhabited cell of semi‐natural/natural vegetation.

## 2 METHODS

### 2.1 Biodiversity data

Details of data collation have been published previously (De Palma et al., 2016; Hudson et al., 2014, 2017) so only a brief description follows. Data were sought from the published literature where bee species were sampled comparably in sites facing different land‐use pressures. We identified suitable papers by searching Web of Science, advertising requests for data and assessing references within relevant reviews; the dataset was further supplemented with the PREDICTS database (www.predicts.org.uk). Criteria for selection were (1) multiple sites (≥2) were sampled for bee abundance or occurrence using the same sampling method within the same season and (2) geographic coordinates were available for each site. Preference was given to studies of sites that were sampled since February 2000, so that diversity data could be matched with remotely sensed data from NASA's Moderate Resolution Imaging Spectroradiometer (Justice et al., 1998). We extracted occurrence and abundance data at each site from suitable papers (hereafter, sources) where possible. Raw data were usually not included within the source or supplementary files so we asked corresponding authors for these data. Some sources report separately data collected in different ways or at different times of year. We term each separate dataset a “study”: within, but not between, studies, diversity data can be compared straightforwardly among sites. Datasets spanning multiple countries were split into separate studies for each country to account for broad‐scale biogeographic variation in diversity. Differences in sampling effort among sites within a study were corrected for, assuming that recorded abundance increases linearly with sampling effort (validated in De Palma et al., 2016). Within each study, we recorded any blocked or split‐plot design. For this analysis, we only used data on species abundances where the entire bee community was sampled, rather than studies where researchers only targeted a single species or a small *a priori* set of species.

### 2.2 Land‐use data

For each site in the dataset, we classified the land use and use intensity based on information in the source, using the scheme described in Hudson et al. (2014, 2017); reproduced in Appendix Table S1.2). Land use was classified as primary vegetation (native vegetation not known to have ever been completely destroyed), secondary vegetation (where the primary vegetation has been completely destroyed; this can include naturally recovering, actively restored sites and semi‐natural sites), cropland (planted with herbaceous crops), plantation forest (planted with crop trees or shrubs), pasture (regularly or permanently grazed by livestock) or urban (areas with human habitation, where vegetation is predominantly managed for civic or personal amenity). The use‐intensity scale is a qualitative, coarse measure of human disturbance (three levels: low, medium and high; Hudson et al., 2014, 2017). For instance, high‐intensity cropland would be monocultures with many signs of intensification (e.g., large fields, high levels of external inputs, irrigation and mechanization); medium‐intensity cropland would show some, but not all, features of higher intensity cropland; low intensity would be small mixed‐cropping fields with little to no external inputs, irrigation or mechanization.

We collapsed levels of land use and intensity when combinations did not have enough data for robust modelling, resulting in the following levels: semi‐natural/natural vegetation (including both primary and secondary vegetation, 121 sites); pasture (76 sites); low‐intensity cropland (208 sites); medium‐intensity cropland (417 sites); high‐intensity cropland (577 sites); and urban (47 sites). We used global layers to estimate, for each site, its distance to the nearest road (Center for International Earth Science Information Network (CIESIN), Columbia University, and Information Technology Outreach Services (ITOS) & University of Georgia, 2013) and the human population density (Balk et al., 2006; Center for International Earth Science Information Network (CIESIN), Columbia University, International Food Policy Research Institute (IFPRI), The World Bank, & Centro Internacional de Agricultura Tropical (CIAT), 2011) for the pixel (30″) containing the site.

### 2.3 Land‐use maps

The land‐use maps were generated by downscaling the harmonized land‐use dataset for 2005 (Hurtt et al., 2011); full methodological details are published in Hoskins et al. (2016). This land‐use map had multiple values per grid square (the percentage covered by each land‐use class) and land‐use classes matched those in our dataset. Agricultural use‐intensity maps (1 km^2^ resolution for the year 2000) were taken from Temme and Verburg (2011). This map had one value per grid square (extensive pasture, intensive pasture, light, moderate or intensive cropland, or other land uses); the cropland categories were matched to our definitions, becoming low‐, medium‐ and high‐intensity cropland. All maps were projected to Alber's equal area projection; the use‐intensity maps were also downscaled to 30″ to match other data, using the nearest‐neighbour method in ArcGIS v10.0. We restricted all datasets to the EU27 region, as the agricultural use‐intensity maps were only available for these countries. We then combined the land use and agricultural intensity maps using the use‐intensity maps to classify the intensity of cropland where grid cells overlapped with the land‐use class maps. Many grid squares contained a very small amount of cropland, but were not classified as such in the use‐intensity maps. We classified these as low intensity; such grid squares generally contained too little agricultural land for PREDICTS to have classified it as high intensity and this treatment will likely produce maps with conservative estimates of diversity loss (see Appendix S3 for maps of cropland intensity).

### 2.4 Trait data

Trait data were collected by SR and MK from a variety of sources, including published and unpublished literature. Morphometric measurements were taken directly from museum specimens. Trait data pertained to flight season, body size (a proxy for foraging distance; Greenleaf, Williams, Winfree, & Kremen, 2007), reproductive strategy, phenology, dietary breadth and nesting strategy (see Appendix Table S1.3 for details). Thirty‐seven species had incomplete data; 13 additional species had no trait data (11.7% and 4.1% of the total species in the dataset respectively). In R (version 3.2.5: R Core Team, 2016), we used missForest (r package version 1.4: Stekhoven & Buhlmann, 2012; Stekhoven, 2013) to impute missing trait information, including phylogenetic eigenvectors (Diniz‐Filho et al., 2012; PVR package Version 0.2.1: Santos, Diniz‐Filho, Rangel, & Bini, 2012) as predictor variables (using the most extensive bee phylogeny published to date: Hedtke, Patiny, & Danforth, 2013). This method is appropriate when using phylogenetic information to impute categorical and continuous traits (Penone et al., 2014), and accuracy was fairly high (the normalized root mean squared prediction error for continuous variables was 0.160, and the proportion of falsely classified categorical variables was 0.042; numbers range from zero to one, from good to poor predictive ability). Functional diversity is likely to be biased towards phylogenetic diversity, relative to what a complete dataset might show, because phylogeny was used in the imputation; however, results were similar when the original, incomplete trait dataset was used in analyses (results not shown).

### 2.5 Phylogenetic tree

We use a recently published phylogeny of bee species that includes some, but not all, of the species in our dataset (Hedtke et al., 2013). If missing species are also phylogenetically distinct, their absence could bias estimates of phylogenetic diversity; species missing from the phylogeny tend also to have more uncertain estimates of response to land‐use pressures (i.e., larger standard errors; De Palma et al., in prep.). We therefore used a birth–death polytomy resolver in R statistical software (pastis package version 0.1–2: Thomas et al., 2013; R Core Team, 2016) and mrbayes (version 3.2 Ronquist et al., 2012) run via CIPRES (Miller, Pfeiffer, & Schwartz, 2010) to place missing species given their taxonomic affinities, producing 1,000 complete trees (See Appendix S2 and Table S2.1 for details). We randomly sampled 100 trees for use in further analyses to ensure that results were robust to species placement.

### 2.6 Response variables

We used Rao's (1982) quadratic entropy (Q), an abundance‐weighted measure of diversity, to calculate the species, functional and phylogenetic diversity of communities:

(1) $$Q=\sum_{i.j}d_{ij}p_{i}p_{j}$$

where *d*_*ij*_ is the distance—species, functional or phylogenetic—between species *i* and *j*;* p*_*i*_ and *p*_*j*_ are their relative abundances. Rao's Q provides a useful consistent framework for assessing and comparing species, functional and phylogenetic diversity: each facet is calculated by applying the equation to the relevant distance matrix. For species diversity, the distance between each species is equal to unity (i.e., each species is considered fully distinct from all others), making Rao's Q equal to the Gini–Simpson index. Functional distances were calculated using Gower's dissimilarity matrix (which allows for both quantitative and qualitative traits; Gower, 1971; Ricotta & Moretti, 2011); the square‐root correction was applied to provide a dissimilarity matrix with Euclidean properties following Debastiani and Pillar (2012, SYNCSA package) and Stuart‐Smith et al. (2013). Following the picante package in R (version 106‐2: Kembel et al., 2010), the phylogenetic dissimilarity between species was half the cophenetic distance (i.e., the mean distance to the nearest common ancestor). We calculated the mean phylogenetic diversity across the sample of 100 trees. All diversity measures were transformed into effective numbers of species to facilitate comparison among dimensions of biodiversity (by expressing the indices in the same units), using the following transformation:

(2) $$E=\;\frac{1}{1-Q}$$

where *Q* is Rao's quadratic entropy as calculated in Equation (1). For species diversity, *E* equates to the inverse Simpson's Index, which can be thought of as the number of common species (Hill, 1973). This transformation was first suggested by Jost (2006) as way of transforming the Gini–Simpson index into effective species numbers, but has since been further developed for Rao's Q (de Bello, Lavergne, Meynard, Lepš, & Thuiller, 2010; Ricotta & Szeidl, 2009) and applied to calculate species, functional (Cisneros, Fagan, & Willig, 2015; Stuart‐Smith et al., 2013) and phylogenetic diversity (Cisneros et al., 2015).

Rao's Q as a functional diversity metric has been shown to respond to environmental change in a number of animal groups and can complement species diversity measures (Vandewalle et al., 2010). The metric is closely related to other functional diversity measures; for instance, it is significantly correlated with functional dispersion (which is unsurprising given its close mathematical relationship: Laliberté & Legendre, 2010) and correlates moderately with Functional Richness (Mouchet, Villéger, Mason, & Mouillot, 2010; proposed by Villéger, Mason & Mouillot, 2008).

### 2.7 Analysis

Using R (version 3.2.5: R Core Team, 2016), we first tested the correlation between species diversity and the other diversity measures among sites within studies. Mixed‐effects models (version 1.1‐12: Bates, Mächler, Bolker, & Walker, 2015) with Gaussian errors were used, including functional or phylogenetic diversity as response variables, ln‐transformed species diversity and UN subregion as the explanatory variables (including interaction), and random effects to account for the non‐independence of data arising from differences in sampling methodology and biogeography (“study”) and the spatial structure of sites (“block”). The best random‐effect structure was first assessed by comparing Akaike's Information Criterion for all possible random effect structures, which included random slopes (within “study”; Zuur, Ieno, Walker, Saveliev, & Smith, 2009). The fixed‐effects structure was simplified (with models fitted using Maximum Likelihood) using backwards stepwise model simplification and likelihood ratio tests, until the minimum adequate model was obtained (Crawley, 2007; Zuur et al., 2009). Such stepwise model simplification performs similarly to alternative approaches (Murtaugh, 2009) and is a simple suitable technique when a few non‐collinear variables are of interest for building a model to assist in both explanation and prediction. We used marginal $R_{\mathrm{GLMM}}^{2}$ values (Nakagawa, Miguchi, & Nakashizuka, 2006; mumin r package version 1.15.6: Barton, 2016) to determine how much variance in functional and phylogenetic diversity was explained by species diversity.

We then analysed species, functional and phylogenetic diversity as a function of land use and intensity, distance to the nearest road and human population density using linear mixed‐effects models with Gaussian errors. UN subregion was considered as a fixed effect to control for differences in diversity across geographic regions. We considered all two‐way interactions between land use and intensity and other variables. Species diversity was ln‐transformed to normalize residuals; model checking showed that our treatment of response variables was appropriate (a Poisson error structure could not be used as the response variable did not consist only of integers). Human population density and distance to roads were log(+1)‐transformed to improve normality and centred to reduce collinearity. Full models were assessed for multicollinearity using generalized variance inflation factors (GVIFs: Zuur et al., 2009), which were all below 4 (indicating acceptable levels of collinearity).

The initial random effects structure and the process for determining the best random and fixed effect structures were as before. Type II anova tables were computed for the final model (car package version 2.1‐2: Fox & Weisberg, 2011), and bootstrapped confidence intervals were calculated from 1000 bootstrap samples (coefficient estimates are considered significant if bootstrapped confidence intervals do not cross zero). Residuals of final models did not show strong evidence of spatial autocorrelation (Moran's I test, Appendix S4.1).

We used the coefficients from these models (fitted using Restricted Maximum Likelihood) to predict the biodiversity in each grid cell, relative to the level of diversity predicted in a grid square consisting entirely of semi‐natural/natural vegetation with a human population density of zero. This approach is similar to that used by Reidsma, Tekelenburg, van den Berg, and Alkemade (2006) to derive a measure of “ecosystem quality,” although their measure relied on dose–response relationships between biodiversity and agricultural intensity synthesized through a literature review, while our approach uses direct analysis of primary data from Europe. We calculate the average species, functional and phylogenetic diversity for each country in the EU27 region, relative to baseline. Responsibility for reporting and acting on biodiversity loss is at the country level so they are a natural and understandable unit of accounting.

We also assess the spatial mismatch between diversity measures in a multistep process using the predicted values of species, functional and phylogenetic diversity from each grid square across the EU27 region. Functional and phylogenetic diversity are expected to be correlated with species diversity (as the chance of a community including different parts of trait or phylogenetic space increase as species diversity increases). If the correlation between diversity measures is perfect, a model of predicted phylogenetic diversity as a function of predicted species diversity for instance will have residuals with values of zero. If however the correlation between measures is less than perfect, positive residuals would indicate that phylogenetic diversity is *higher* than expected based on species diversity alone, and negative residuals would indicate the opposite. Mapping these residuals shows the spatial pattern in these mismatches. We show this spatial mismatch between diversity measures by first modelling predicted functional and phylogenetic diversities against predicted species diversity using ordinary least‐squares regression and mapping the two sets of residuals (to show where the losses in the two diversity measures vary independently from species diversity losses). We then mapped the residuals from a further model relating these two residual maps, to assess the spatial mismatch between losses in functional and phylogenetic diversity, after correlations with species diversity had been accounted for.

## 3 RESULTS

Among sites within studies, species diversity explained over 75% of the variation in functional (χ^2^ = 877.49, *df *= 1, *p *<* *.0001) and phylogenetic diversity (χ^2^ = 838.05, *df *= 1, *p *<* *.0001). There were positive relationships between diversity measures, although the slopes varied among studies. Fixed and random effects together explained over 85% of the variation in functional and phylogenetic diversity.

**Figure 1:**
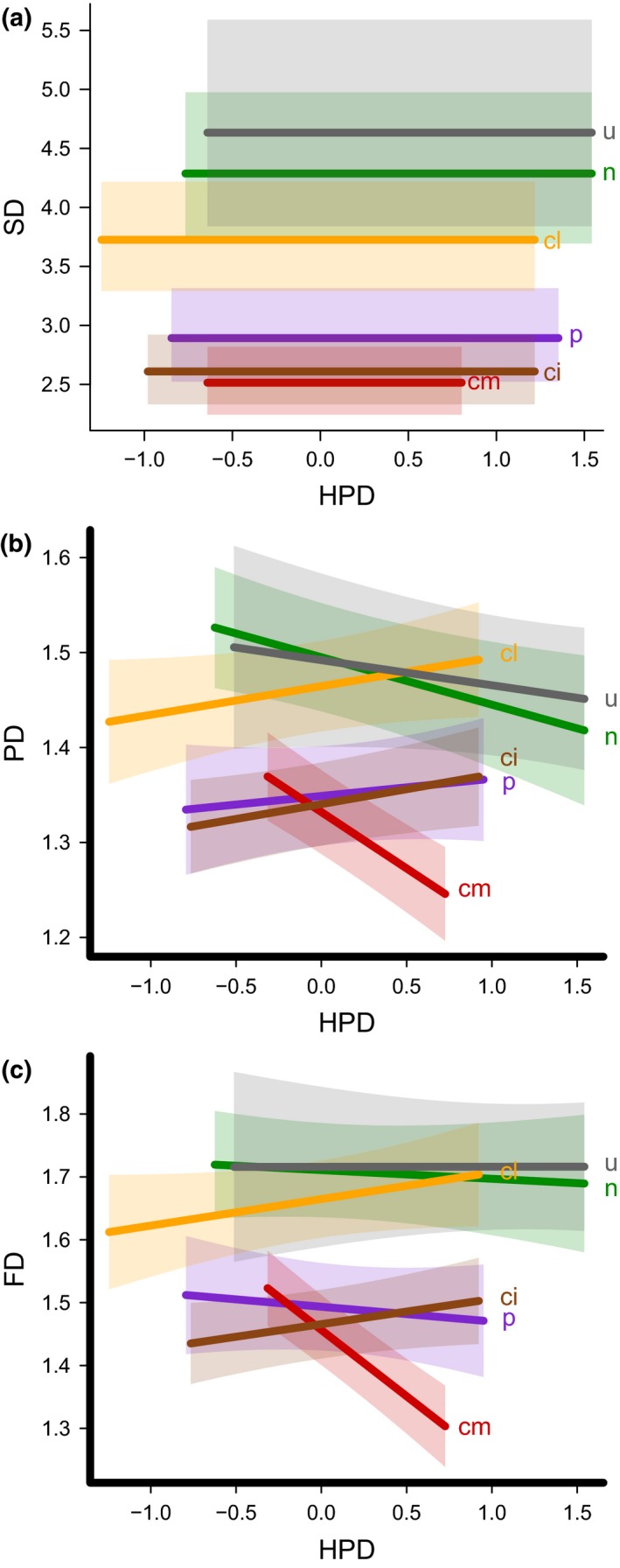
Relationship of (a) Species diversity (SD), (b) Phylogenetic diversity (PD) and (c) Functional diversity (FD) with log‐transformed and centred human population density (HPD) in different land uses, ± one standard error. n, semi‐natural/natural vegetation; p, pasture; cl, low‐intensity cropland; cm, medium‐intensity cropland, ci, high‐intensity cropland; u, urban. Note that for SD, there was no significant relationship with HPD so flat lines are presented. [Colour figure can be viewed at wileyonlinelibrary.com]

**Figure 2:**
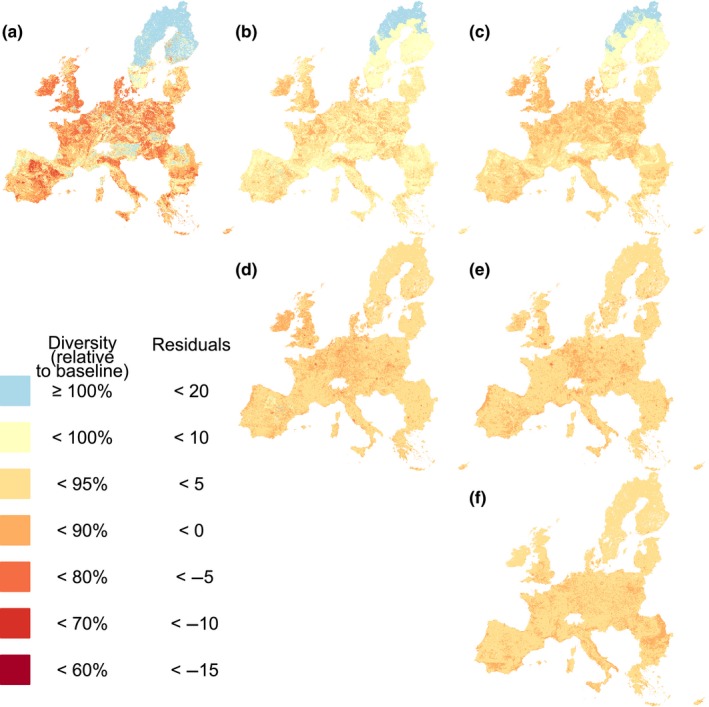
Maps of (a) taxonomic, (b) functional and (c) phylogenetic diversity relative to the baseline: the expected diversity if the grid cell was entirely covered with natural and semi‐natural vegetation, with 0 human population density. A value of 100% would therefore indicate that the grid cell has a level of diversity equivalent to the baseline; numbers below 100% indicate a loss in diversity relative to the baseline. Panel (d) shows the residuals of a model between species diversity (a) and functional diversity (b); panel (e) shows the residuals of a model between species diversity (a) and phylogenetic diversity (c); panel (f) shows the residuals of a model between the maps in panels (d) and (e). These maps were produced for the EU27 region using Alber's equal area conical projection (resolution of 30 arc‐seconds). [Colour figure can be viewed at wileyonlinelibrary.com]

Species, functional and phylogenetic diversity were all significantly related to land use and intensity (Figure 1; Appendix Tables S4.1, S4.2 and S4.3). Relative to semi‐natural/natural vegetation, species diversity was reduced by over 30% in pasture (estimate = −0.39, bootstrapped confidence intervals, bCI = −0.68, −0.10) and by over 40% in medium‐intensity cropland (estimate = −0.50, bCI = −0.85, −0.17) and high‐intensity cropland (estimate = −0.48, bCI = −0.82, −0.12; Figure 1). The responses of functional and phylogenetic diversity to land use and intensity were more complex and depended on the level of human population density (Figure 2; functional diversity: χ^2^ = 15.37, *p *<* *.01; phylogenetic diversity: χ^2^ = 12.74, *p *<* *.05). Functional diversity declined significantly more strongly with human population density in medium‐intensity cropland than in more natural land uses (estimate = −3.40, bCI = −6.71, −0.19). At mean levels of human population density, functional and phylogenetic diversity were significantly lower in pasture and both medium‐ and high‐intensity cropland relative to semi‐natural/natural vegetation (Appendix Table S4.2 and S4.3). Distance to roads was not retained in any of the models.

Inferred losses showed particularly weak spatial congruence between species diversity and functional diversity (Figure 2d); species diversity losses were up to 48 percentage points (pp.) more severe and up to 18 pp. less severe than functional diversity losses. Similarly, the loss of species diversity was up to 38 pp. more severe and 18 pp. less severe than losses in phylogenetic diversity (Figure 2e). In particular, urban areas that showed high species diversity (relative to an unimpacted baseline) tended to have losses in other aspects of diversity. The differences between functional and phylogenetic diversity losses were less marked, but were still evident after accounting for correlations with species diversity (Figure 2f).

The average losses of local diversity varied considerably across countries (Table 1). Average losses of local functional and phylogenetic diversity were much lower than for species diversity. Even so, most countries have lost over 5% of their diversity (24/27 countries for functional diversity, and 25/27 countries for species and phylogenetic diversity).

**Table 1:** Diversity relative to baseline in each EU27 country. The baseline (100) represents the expected diversity if the entire grid square consisted of semi‐natural or natural vegetation with 0 human population density. Note that the standard errors incorporate only spatial variation in the maps, not the underlying uncertainty in model coefficients or structure. SD, species diversity; FD, functional diversity; PD, phylogenetic diversity

Country	TD relative to baseline (±standard error)	FD relative to baseline (±standard error)	PD relative to baseline (±standard error)
Austria	92 (±0.03)	94.2 (±0.01)	92.6 (±0.01)
Belgium	87.4 (±0.04)	92.6 (±0.01)	90.4 (±0.01)
Bulgaria	88.2 (±0.02)	93.8 (±0.01)	91.6 (±0.01)
Cyprus	87.6 (±0.06)	93.1 (±0.03)	91 (±0.02)
Czech Republic	86.5 (±0.03)	92.8 (±0.01)	90.8 (±0.01)
Hungary	82 (±0.03)	92 (±0.01)	89.7 (±0.01)
Lithuania	88.5 (±0.02)	93.8 (±0.01)	91.6 (±0.01)
Latvia	90.7 (±0.02)	94.5 (±0.01)	92.9 (±0.01)
Luxembourg	89.9 (±0.11)	93.6 (±0.03)	91.4 (±0.03)
Netherlands	85.1 (±0.04)	91.8 (±0.01)	89.6 (±0.01)
Poland	84.2 (±0.02)	92.4 (±0)	90.3 (±0)
Malta	91.4 (±0.18)	95.2 (±0.12)	91.7 (±0.07)
Romania	89.7 (±0.01)	94.2 (±0)	91.9 (±0)
Slovakia	88.7 (±0.04)	93.4 (±0.01)	91.6 (±0.01)
Slovenia	93.2 (±0.05)	94.7 (±0.02)	93.2 (±0.02)
Germany	86.3 (±0.01)	92.2 (±0)	90.2 (±0)
Denmark	79.7 (±0.04)	90.7 (±0.01)	88.9 (±0.01)
Estonia	91.5 (±0.02)	94.5 (±0.01)	93.1 (±0.01)
Spain	85.9 (±0.01)	93.3 (±0)	91.2 (±0)
Finland	98.6 (±0.01)	98 (±0)	97.4 (±0)
France	86.7 (±0.01)	92.9 (±0)	91 (±0)
United Kingdom	83.2 (±0.02)	91.2 (±0.01)	89.6 (±0)
Greece	86.9 (±0.02)	93.1 (±0.01)	91.3 (±0.01)
Ireland	80.5 (±0.02)	89.8 (±0.01)	88.5 (±0.01)
Italy	87.5 (±0.01)	93.1 (±0.01)	91 (±0)
Sweden	98.1 (±0.01)	97.8 (±0)	97.1 (±0)
Portugal	90.7 (±0.02)	93.9 (±0.01)	92 (±0.01)

## 4 DISCUSSION

Bee species' responses to human impacts are extremely varied (Cariveau & Winfree, 2015); while some species benefit from human‐dominated land uses, others are intolerant of land‐use change (Banaszak‐Cibicka & Zmihorski, 2012; Bates et al., 2011; De Palma et al., 2015). Our work shows that despite this heterogeneity, bee community diversity is significantly reduced in pasture and higher intensity cropland relative to semi‐natural/natural vegetation, while urban areas tend to maintain diversity (although the sample size was relatively small). This finding may partly explain why heterogeneity in species' responses may not always stabilize pollination service provision, as diversity losses in the short‐term are not necessarily counterbalanced by gains (Cariveau, Williams, Benjamin, & Winfree, 2013). Our results suggest that abundant, common species—probably the most economically important for pollination service provision (Kleijn et al., 2015; Winfree, Fox, Williams, Reilly, & Cariveau, 2015)—are responding negatively to human impacts, as the abundance‐weighted diversity measures used here are most influenced by dominant species (de Bello, Lepš, Lavorel, & Moretti, 2007; Leinster & Cobbold, 2012).

### 4.1 Responses of bee diversity to land use and related pressures

Bee species diversity was significantly reduced in pasture and more intensively managed cropland, but not in low‐intensity cropland. Additionally, there was no strong relationship between diversity and human population density. These results suggest on average a conservation benefit of low‐input cropland, with relatively high diversity even in more densely populated areas. Previous research has also found that less intensive agricultural practices can result in higher species (Gabriel, Sait, Kunin, & Benton, 2013; Tuck et al., 2014) and functional richness (Rader, Bartomeus, Tylianakis, & Laliberté, 2014) of bees, particularly where the surrounding landscape is of low quality (Kennedy et al., 2013).

Urban areas also maintained relatively high diversity, even at higher population densities. Although our dataset included relatively few urban sites, this result is consistent with Baldock et al. (2015), who found fairly high bee diversity in urban areas compared to farmland and nature reserves. Urban areas can support diverse communities, including some specialized species, as the presence of exotic plants can lengthen the nectar season (Salisbury et al., 2015).

Higher human population density in the surrounding landscape significantly influenced functional and phylogenetic diversity in different land uses. It reduced the functional diversity found in medium‐intensity (but not high‐intensity) cropland significantly more than in semi‐natural/natural vegetation. It is possible that in high‐intensity cropland, which has experienced strong ecological filtering of species (De Palma et al., 2015; Rader et al., 2014), only resistant species now remain, such that increasing human population density in the surrounding landscape has little influence. In contrast, medium‐intensity cropland may show weaker filtering effects (De Palma et al., 2015) such that more sensitive species still remain. For example, Carré et al. (2009) found that species that were positively associated with cropland and urban areas were generally less sensitive to landscape change.

Though the mechanism for the complex relationship between diversity and human population density is unclear, these results suggest that although urban areas may help maintain species diversity, functional diversity in the surrounding landscape may be at risk, even where agricultural practices are less intensive. These findings are particularly important as urbanization will probably be a strong driver of changes to biodiversity and ecosystem service provision in Europe (Eigenbrod et al., 2011). Sprawling urbanization could reduce agricultural productivity (Eigenbrod et al., 2011); we can speculate from our results that human population growth in agricultural areas could also be more detrimental to bee diversity than increasing the density of existing urban areas (Sushinsky, Rhodes, Possingham, Gill, & Fuller, 2013), potentially further reducing agricultural productivity.

The different responses of species, functional and phylogenetic diversity to land‐use pressures highlight the importance of assessing multiple facets of diversity. Focusing only on species diversity may be appropriate for some conservation questions, especially where the aim is to protect diversity for its inherent value. However, if the aim is to better characterize risks to and identify conservation priorities for securing an efficient pollination service, then focusing only on species diversity may at best be insufficient: species diversity sometimes shows a less extreme response than functional diversity but functional diversity may be more important than species diversity for pollination service provision (e.g. Hoehn et al., 2008). At worst—where species diversity shows different responses from functional diversity—a focus on species diversity may be misleading, resulting in conservation prioritizations that are incorrect given the aim.

### 4.2 Losses of local bee diversity across Europe

The significant effects of land use, cropland intensity and human population density on bee diversity suggest strong spatial patterns of loss across Europe, relative to the baseline (the estimated diversity in uninhabited semi‐natural/natural vegetation). Over half of countries across the region are estimated to have lost over 10% of their average local species diversity. Countries in Western Europe—where much of the land is covered by intensive cropland and pasture—have seen particularly high impacts, whereas Sweden and Finland were generally only minimally impacted. (N.B. We do not attempt to estimate changes in overall gamma diversity in countries or across Europe.) However, it is unclear whether there is a threshold of bee diversity beyond which pollination services are threatened or at what spatial scale such thresholds are most relevant (Brook, Ellis, Perring, Mackay, & Blomqvist, 2013; Mace et al., 2014).

### 4.3 Congruence between different facets of diversity

The losses in species diversity tended to be more extreme than losses in functional or phylogenetic diversity. This tendency is expected because only the measure of species diversity assumes that all species are maximally (and equally) dissimilar to one another; thus, there is redundancy among species' contributions to functional and phylogenetic—but not species—diversity. Nee and May (1997) showed that among species redundancy in phylogenetic diversity (caused by the hierarchical nature of phylogenies) means that diversity can be relatively well maintained even when most species are lost.

Importantly, although species diversity explained over 75% of the within‐study variation in functional and phylogenetic diversity, the three measures did not always respond similarly to land‐use impacts. Therefore, while different facets of diversity are strongly related (Stevens & Tello, 2014), species diversity is not an effective surrogate for functional or phylogenetic diversity when considering responses to human impacts (Cisneros et al., 2015). This has implications for how conservation actions are prioritised and evaluated, as species‐diverse assemblages may be phylogenetically and functionally impoverished; in some areas, losses in phylogenetic diversity were almost 20 percentage points greater than species diversity losses.

Losses of functional and phylogenetic diversity were relatively congruent, although functional diversity losses were slightly less extreme. As trait data are still incomplete and time‐consuming to obtain, these results imply that phylogenetic diversity may provide an efficient, effective alternative to assessing functional diversity (Faith, 2013), even with incomplete genetic information. However, two caveats are important here. First, functional and phylogenetic community diversity were not fully independent. Second, functional and phylogenetic diversity can show incongruent patterns of loss if the traits‐mediating species' sensitivity show strong phylogenetic signal (Fritz & Purvis, 2010).

### 4.4 Limitations of the study

Our analysis uses spatial comparisons to determine diversity loss across different land‐use classes. Because we do not have historic data on species abundances before land‐use conversion, our “baseline” levels of diversity are instead estimated from primary and secondary vegetation remnants, often in highly fragmented, agricultural landscapes. These sites are likely to show some shift in diversity relative to the past, due to human activities both inside and around these sites; for example, many pollinator species have already been driven to regional extinction by human activities (Ollerton, Erenler, Edwards, & Crockett, 2014). Indeed, despite our dataset including almost 1,500 sites across Europe, less than one fifth of Europe's bee species were recorded, with biases towards genera that are species rich (such as *Andrena* and *Lasioglossum*) and readily identifiable in the field (e.g. *Bombus*). Conversely, we may have overestimated the benefit of primary vegetation for bee diversity. In temperate regions, wooded areas are often not particularly beneficial for bees (Winfree, Griswold, & Kremen, 2007), so diversity can increase with low amounts of disturbance that open up habitats or with low‐input agricultural activities that can provide floral and nesting resources; this is especially true in a region such as Europe, which has had a long history of agricultural use, to which many species will have adapted. However, our sample size for primary vegetation was too small to estimate diversity in these areas separately.

It is also possible that the impact of recent land‐use change has not yet been fully realized due to biotic lag (Essl et al., 2015); for instance, extinction lags have been supported for a number of taxa in Europe, including dragonflies, grasshoppers and plants (Dullinger et al., 2013). Bee communities may therefore still be responding to recent shifts to improve the legal status of protected areas (e.g., Natura 2000) and widespread implementation of pollinator‐friendly agri‐environment scheme options over the last two decades; however, the rate of species richness loss and biotic homogenization appear to have already slowed in Europe (Carvalheiro et al., 2013).

Our cropland intensity classification is coarse; although levels of external inputs contribute to the classification, we do not consider the make‐up of these inputs, changes to which could have greater impacts on beneficial insects than changes in the amount (e.g., neonicotinoids can have adverse impacts on bee colonies and populations: Henry et al., 2012; Whitehorn, O'Connor, Wackers, & Goulson, 2012; Woodcock et al., 2016). The fact that our coarse measure still showed a significant impact on all aspects of bee diversity suggests we may have underestimated the true impact of intensification.

While our focus on European bees provided a rich dataset for analysis, it also limits the ability to generalize our results as bee communities can respond differently across regions (De Palma et al., 2016). Process‐based explanations for the observed patterns of diversity loss are beyond the scope of our study, but would provide a firmer basis for generalization and extrapolation (Evans et al., 2013).

## 5 CONCLUSION

We have provided the first continental‐scale assessment of how species, functional and phylogenetic diversity of European bee communities are impacted by land‐use class, cropland intensity and human population density. We show that responses to human impacts—and estimated losses across Europe—are not equivalent or even fully redundant across these facets of diversity, highlighting the need to explore multiple diversity measures for a comprehensive understanding of biodiversity loss and potential implications for ecosystem functioning. As our study is limited to Europe—mainly a highly altered, intensively managed landscape—our conclusions are unlikely to be generalizable to other less altered systems (De Palma et al., 2016; Winfree, 2013); further collation of trait, genetic and assemblage data on bee species across the globe will be necessary to explore local diversity losses in other regions.

## BIOSKETCH

**Adriana De Palma** is a post‐doctoral researcher at the Natural History Museum in London. Her primary research interest is using data synthesis to understand the spatial and temporal responses of biodiversity to anthropogenic change, with a particular focus on insect pollinators. Adriana works with the PREDICTS project—Projecting Responses of Ecological Diversity in Changing Terrestrial Systems (http://www.predicts.org.uk/)—which is led by Andy Purvis.

Author contributions: A.D.P. and A.P. designed the study; M.K., R.B., S.G.P., S.P.M.R. and O.S. made substantial data contributions; A.D.P. led the analysis; all authors contributed greatly to the interpretation of results; A.D.P. wrote the first draft of this manuscript and all authors contributed to the final version.
