# Supplementary material for: Dimensions of biodiversity loss: Spatial mismatch in land‐use impacts on species, functional and phylogenetic diversity of European bees
Source: Divers Distrib. 2017 Sep 13;23(12):1435–46. doi: 10.1111/ddi.12638 (PMC5699437; doi:10.1111/ddi.12638)
Supplement: Supplementary file 1 [file DDI-23-1435-s001.pdf]

Supplementary Material: Dimensions of biodiversity loss: spatial mismatch in land-use impacts on species, functional and phylogenetic diversity of European bees

De Palma et al

Appendix S1 Dataset collation

Table S1.1: The following are data sources used in this analysis. See De Palma *et al.* (2016) for full details of dataset compilation. †Data are taken from the PREDICTS database (openly available via `http://data.nhm.ac.uk`; Hudson *et al.*, 2017). ‡Data are available from the referenced paper. For all other datasets, please contact the corresponding author of that paper directly.

Source Number	Reference
1	Kruess & Tscharntke (2002)
2	Quaranta *et al.* (2004)†
3	Marshall *et al.* (2006)†
4	Meyer *et al.* (2007)†
5	Billeter *et al.* (2007)† , Diekötter *et al.* (2008)† and Le Féon *et al.* (2010)†
6	Franzén & Nilsson (2008)†

7	Kohler *et al.* (2007)†
8	Albrecht *et al.* (2010)
9	Bates *et al.* (2011)†
10	Power & Stout (2011)†
11	Samnegård *et al.* (2011)†
12	Schüepp *et al.* (2011)†
13	Weiner *et al.* (2011)
14	Hanley (2011)†
15	Mudri-Stojnic *et al.* (2012)†‡
16	Jauker *et al.* (2013); Meyer *et al.* (2009)†
17	Fowler (2014)†

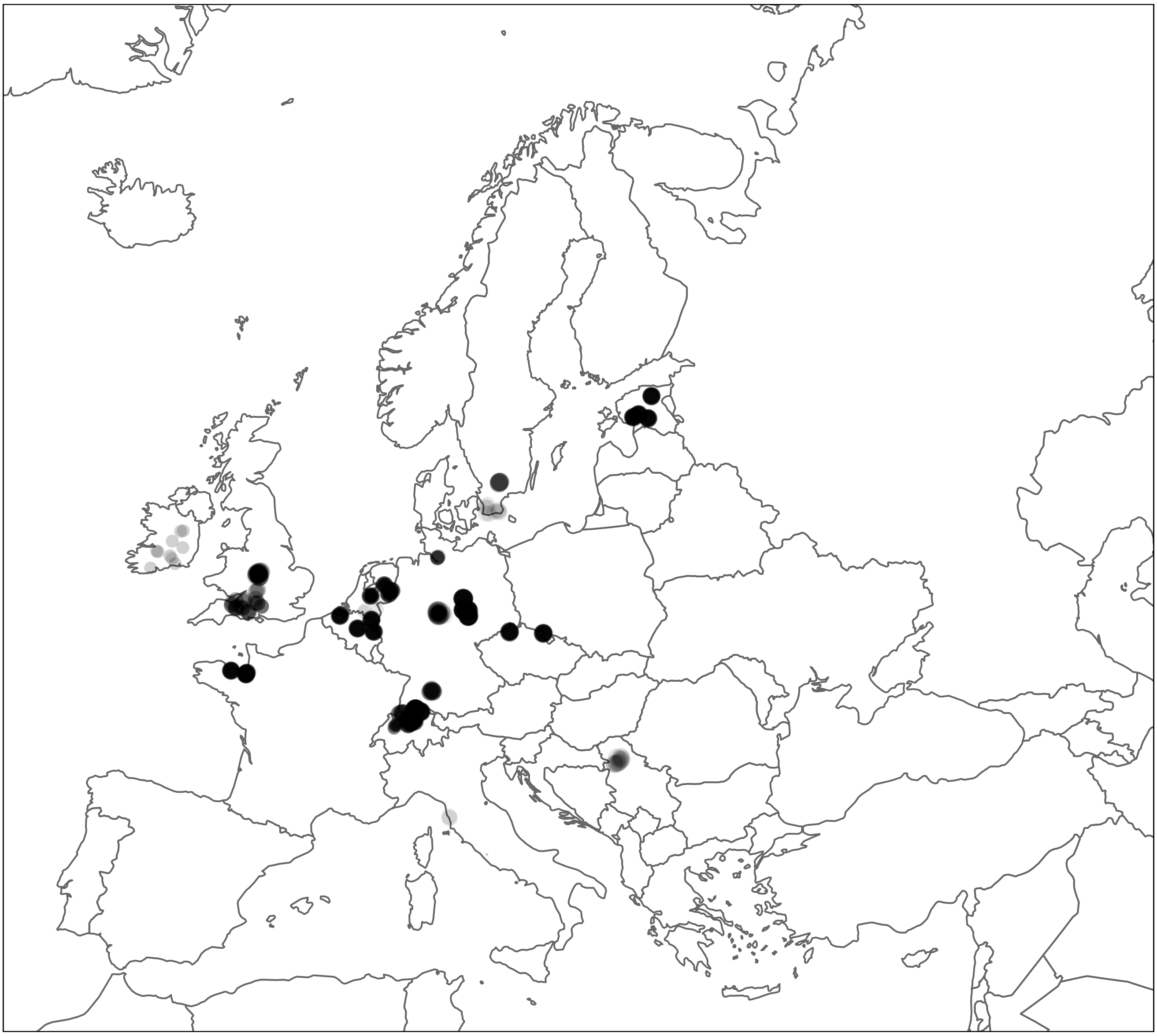

Figure S1.1: Map of sites in Europe from which bee abundance data were available. The size of the points correspond to the number of bee species sampled at the site (log-transformed). Note that all points are semi-transparent; points that appear opaque are therefore indicative of areas where multiple sites have been sampled.

Table S1.2: Land-use class and intensity definitions as used in Hudson *et al.* (2014)

Level 1 Land Use	Predominant Land Use	Minimal use	Light use	Intense use
**No evidence of prior destruction of the vegetation**	**Primary forest**	Any disturbances identified are very minor (e.g., a trail or path) or very limited in the scope of their effect (e.g., hunting of a particular species of limited ecological importance).	One or more disturbances of moderate intensity (e.g., selective logging) or breadth of impact (e.g., bushmeat extraction), which are not severe enough to markedly change the nature of the ecosystem. Primary sites in suburban settings are at least Light use.	One or more disturbances that is severe enough to markedly change the nature of the ecosystem; this includes clear-felling of part of the site too recently for much recovery to have occurred. Primary sites in fully urban settings should be classed as Intense use.
	**Primary Non-Forest**	As above	As above	As above
**Recovering after destruction of the vegetation**	**Mature Secondary Vegetation**	As for Primary Vegetation-Minimal use	As for Primary Vegetation-Light use	As for Primary Vegetation-Intense use
	**Intermediate Secondary Vegetation**	As for Primary Vegetation-Minimal use	As for Primary Vegetation-Light use	As for Primary Vegetation-Intense use
	**Young Secondary Vegetation**	As for Primary Vegetation-Minimal use	As for Primary Vegetation-Light use	As for Primary Vegetation-Intense use
	**Secondary Vegetation (indeterminate age)**	As for Primary Vegetation-Minimal use	As for Primary Vegetation-Light use	As for Primary Vegetation-Intense use

**Human use (agricultural)**	**Plantation forest**	Extensively managed or mixed timber, fruit/coffee, oil-palm or rubber plantations in which native understorey and/or other native tree species are tolerated, which are not treated with pesticide or fertiliser, and which have not been recently ($<$ 20 years) clear-felled.	Monoculture fruit/coffee/rubber plantations with limited pesticide input, or mixed species plantations with significant inputs. Monoculture timber plantations of mixed age with no recent ($<$ 20 years) clear-felling. Monoculture oil-palm plantations with no recent ($<$ 20 years) clear-felling.	Monoculture fruit/coffee/rubber plantations with significant pesticide input.Monoculture timber plantations with similarly aged trees or timber/oil-palm plantations with extensive recent ($<$ 20 years) clear-felling.
	**Cropland**	Low-intensity farms, typically with small fields, mixed crops, crop rotation, little or no inorganic fertiliser use, little or no pesticide use, little or no ploughing, little or no irrigation, little or no mechanisation.	Medium intensity farming, typically showing some but not many of the following: large fields, annual ploughing, inorganic fertiliser application, pesticide application, irrigation, no crop rotation, mechanisation, monoculture crop. Organic farms in developed countries often fall within this category, as may high-intensity farming in developing countries.	High-intensity monoculture farming, typically showing many of the following features: large fields, annual ploughing, inorganic fertiliser application, pesticide application, irrigation, mechanisation, no crop rotation.
	**Pasture**	Pasture with minimal input of fertiliser and pesticide, and with low stock density (*not* high enough to cause significant disturbance or to stop regeneration of vegetation).	Pasture either with significant input of fertiliser or pesticide, or with high stock density (high enough to cause significant disturbance or to stop regeneration of vegetation).	Pasture with significant input of fertiliser or pesticide, *and* with high stock density (high enough to cause significant disturbance or to stop regeneration of vegetation).
**Human use (urban)**	**Urban**	Extensive managed green spaces; villages.	Suburban (e.g. gardens), or small managed or unmanaged green spaces in cities.	Fully urban with no significant green spaces.

Table S1.3: Original and coarsened factor levels of species traits

Trait	Coarsened factor levels	Original levels	Rationale
Nesting trait	Excavators	Excavators in the soil or vegetation	This trait was coarsened to represent two distinct nesting strategies: those that build their own holes versus those that don't. Excavators are particular about nesting sites, often requiring hard, bare ground or pithy stems, whilst those that don't excavate use existing cavities or old nesting sites, regardless of nest location.
	Non-excavators	Carder bees, renters, masons, cleptoparasites and social parasites	
Sociality	Obligately solitary	Solitary, solitary or communal, communal, cleptoparasitic	The sociality of the species was defined according to how their offspring are raised, because this relates to reproductive capacity. Social species, or those that raise their young in social nests (such as social parasites), are able to produce greater numbers of offspring because there are more workers to provision those offspring. Primitively eusocial species are able to adjust their reproductive capacity, often according to resource requirements: for example, *Halictus rubicundus* is social in warmer, more resource rich areas but solitary in other areas.
	Not obligately solitary	Highly eusocial, primitively eusocial, solitary/primitively eusocial, polymorphic, social parasites	
Lecty	No Lecty status	No Lecty status	Species with no lecty status are those which do not collect their own pollen, for example cleptoparasites. Phenotypic flexibility can be considered as a form of generalism so species that can be either oligolectic or polylectic are considered in the same category as the pollen generalists.
	Obligately oligolectic	Oligolectic	
	Polylectic/Flexible	Polylectic, oligolectic or polylectic	
Voltinism	Univoltine	Univoltine	Species were split into two categories: those with only one generation per year, and those that do have or can have more than one generation per year, as the latter are predicted to be less impacted by local threats
	Multivoltine/Flexible	Bivoltine, multivoltine, univoltine or bivoltine, univoltine or multivoltine	

Appendix S1.1 Species list, based on taxonomy from Michener (2000)

Amegilla quadrifasciata

Andrena agilissima

Andrena alfkenella

Andrena angustior

Andrena anthrisci

Andrena barbilabris

Andrena bicolor

Andrena bucephala

Andrena carantonica

Andrena chrysopus

Andrena chrysosceles

Andrena cineraria

Andrena cinerea

Andrena clarkella

Andrena coitana

Andrena combinata

Andrena congruens

Andrena curvungula

Andrena decipiens

Andrena denticulata

Andrena distinguenda

Andrena dorsata

Andrena enslinella

Andrena falsifica

Andrena flavipes

Andrena florea

Andrena floricola

Andrena florivaga

Andrena fucata

Andrena fulva

Andrena fulvago

Andrena fulvata

Andrena fulvida

Andrena fuscipes

Andrena gravida

Andrena haemorrhoa

Andrena hattorfiana

Andrena helvola

Andrena humilis

Andrena intermedia

Andrena labialis

Andrena labiata

Andrena lagopus

Andrena lapponica

Andrena lathyri

Andrena minutula

Andrena minutuloides

Andrena miserabilis

Andrena mitis

Andrena nasuta

Andrena nigroaenea

Andrena nigroolivacea

Andrena nitida

Andrena nitidiuscula

Andrena niveata

Andrena ovatula

Andrena pandellei

Andrena pilipes

Andrena praecox

Andrena propinqua

Andrena proxima

Andrena rosae

Andrena rufa

Andrena ruficrus

Andrena sabulosa

Andrena saundersella

Andrena semilaevis

Andrena similis

Andrena strohmella

Andrena subopaca

Andrena synadelpha

Andrena tarsata

Andrena tibialis

Andrena ungeri

Andrena vaga

Andrena varians

Andrena ventralis

Andrena viridescens

Andrena vulpecula

Andrena wilkella

Anthidiellum strigatum

Anthidium manicatum

Anthidium punctatum

Anthophora aestivalis

Anthophora atroalba

Anthophora dispar

Anthophora furcata

Anthophora plumipes

Anthophora retusa

Apis mellifera

Bombus barbutellus

Bombus bohemicus

Bombus campestris

Bombus cryptarum

Bombus distinguendus

Bombus gerstaeckeri

Bombus hortorum

Bombus humilis

Bombus hypnorum

Bombus jonellus

Bombus lapidarius

Bombus lucorum

Bombus magnus

Bombus monticola

Bombus muscorum

Bombus norvegicus

Bombus pascuorum

Bombus pomorum

Bombus pratensis

Bombus pratorum

Bombus ruderarius

Bombus ruderatus

Bombus rupestris

Bombus schrencki

Bombus semenoviellus

Bombus soroeensis

Bombus subterraneus

Bombus sylvarum

Bombus sylvestris

Bombus terrestris

Bombus vestalis

Bombus veteranus

Bombus wurflenii

Camptopoeum frontale

Ceratina cucurbitina

Ceratina cyanea

Ceratina nigrolabiata

Chelostoma campanularum

Chelostoma distinctum

Chelostoma florisomne

Chelostoma rapunculi

Coelioxys inermis

Coelioxys rufescens

Colletes cunicularius

Colletes daviesanus

Colletes floralis

Colletes succinctus

Dasypoda plumipes

Dufourea dentiventris

Eucera clypeata

Eucera eucnemidea

Eucera longicornis

Eucera nigrescens

Halictus confusus

Halictus gemmeus

Halictus kessleri

Halictus langobardicus

Halictus maculatus

Halictus patellatus

Halictus pollinosus

Halictus quadricinctus

Halictus rubicundus

Halictus scabiosae

Halictus semitectus

Halictus simplex

Halictus subauratus

Halictus tumulorum

Heriades crenulatus

Heriades truncorum

Hoplitis anthocopoides

Hoplitis claviventris

Hoplitis leucomelana

Hylaeus angustatus

Hylaeus annularis

Hylaeus brevicornis

Hylaeus communis

Hylaeus confusus

Hylaeus difformis

Hylaeus gibbus

Hylaeus gredleri

Hylaeus hyalinatus

Hylaeus nigritus

Hylaeus paulus

Hylaeus punctatus

Hylaeus rinki

Hylaeus signatus

Hylaeus sinuatus

Hylaeus styriacus

Hylaeus variegatus

Lasioglossum aeratum

Lasioglossum albipes

Lasioglossum albocinctum

Lasioglossum brevicorne

Lasioglossum calceatum

Lasioglossum clypeare

Lasioglossum corvinum

Lasioglossum costulatum

Lasioglossum cupromicans

Lasioglossum discum

Lasioglossum fratellum

Lasioglossum fulvicorne

Lasioglossum glabriusculum

Lasioglossum interruptum

Lasioglossum laevigatum

Lasioglossum laticeps

Lasioglossum lativentre

Lasioglossum leucopus

Lasioglossum leucozonium

Lasioglossum lineare

Lasioglossum lucidulum

Lasioglossum majus

Lasioglossum malachurum

Lasioglossum minutissimum

Lasioglossum minutulum

Lasioglossum morio

Lasioglossum nigripes

Lasioglossum nitidiusculum

Lasioglossum nitidulum

Lasioglossum pallens

Lasioglossum parvulum

Lasioglossum pauperatum

Lasioglossum pauxillum

Lasioglossum politum

Lasioglossum punctatissimum

Lasioglossum puncticolle

Lasioglossum pygmaeum

Lasioglossum quadrinotatum

Lasioglossum rufitarse

Lasioglossum sabulosum

Lasioglossum semilucens

Lasioglossum sexnotatum

Lasioglossum sexstrigatum

Lasioglossum smeathmanellum

Lasioglossum subfasciatum

Lasioglossum tricinctum

Lasioglossum villosulum

Lasioglossum xanthopus

Lasioglossum zonulum

Lithurgus cornutus

Macropis europaea

Macropis fulvipes

Megachile albisecta

Megachile alpicola

Megachile apicalis

Megachile centuncularis

Megachile circumcincta

Megachile ericetorum

Megachile ligniseca

Megachile nigriventris

Megachile pilidens

Megachile versicolor

Megachile willughbiella

Melecta albifrons

Melitta haemorrhoidalis

Melitta leporina

Melitta nigricans

Melitta tricincta

Melitturga clavicornis

Nomada alboguttata

Nomada armata

Nomada bifasciata

Nomada castellana

Nomada fabriciana

Nomada ferruginata

Nomada flava

Nomada flavoguttata

Nomada flavopicta

Nomada fucata

Nomada fulvicornis

Nomada goodeniana

Nomada guttulata

Nomada hirtipes

Nomada lathburiana

Nomada leucophthalma

Nomada marshamella

Nomada miniuscula

Nomada obtusifrons

Nomada panzeri

Nomada ruficornis

Nomada rufipes

Nomada sexfasciata

Nomada sheppardana

Nomada signata

Nomada stoeckherti

Nomada striata

Nomada succincta

Nomada zonata

Osmia adunca

Osmia aurulenta

Osmia bicolor

Osmia bicornis

Osmia brevicornis

Osmia caerulescens

Osmia cornigera

Osmia leaiana

Osmia parietina

Osmia spinulosa

Osmia uncinata

Panurgus banksianus

Panurgus calcaratus

Pseudapis diversipes

Rhodanthidium septemdentatum

Rophites canus

Rophites quinquespinosus

Sphecodes albilabris

Sphecodes alternatus

Sphecodes crassus

Sphecodes ephippius

Sphecodes ferruginatus

Sphecodes geoffrellus

Sphecodes gibbus

Sphecodes hyalinatus

Sphecodes miniatus

Sphecodes monilicornis

Sphecodes niger

Sphecodes pellucidus

Sphecodes rufiventris

Sphecodes scabricollis

Sphecodes spinulosus

Tetralonia macroglossa

Tetraloniella alticincta

Tetraloniella lyncea

Tetraloniella nana

Tetraloniella scabiosae

Trachusa byssina

Xylocopa violacea

Appendix S2 Supplementary Methods, Phylogeny

We used a birth-death polytomy resolver (?) to estimate the placement of missing species given their taxonomic affinities: congeners were constrained to be a sister in a monophyletic clade, unless the phylogenetic tree provided evidence against genus monophyly. Where species did not have congeners in the phylogenetic tree, we used higher-level taxonomic constraints for species placement; we only used such constraints where nodes had greater than 95% bootstrap support. *Caenaugochlora*, *Chlerogella*, *Pereirapis*, *Pseudaugochlora*, *Chalepogenus* and *Agapostemonoides* were restricted to their respective tribes, where monophyly was strongly supported (100% bootstrap support, Hedtke *et al.*, 2013). Note that *Agapostemonoides* was constrained within the tribe Caenohalictini, which is sometimes considered only a subtribe within the Halictini tribe (Danforth *et al.*, 2008). *Pachyprosopis* (Euryglossinae: Colletidae) was constrained to be sister to the tribes Euryglossinae, Scrapterinae, and Xeromelissinae, but species were not permitted to enter the clades formed by the Xeromelissinae or Hylaeinae (Almeida & Danforth, 2009; Hedtke *et al.*, 2013). The genus *Ceylalictus* was constrained to be placed within its subfamily, Nomiodinae. Where synonyms were identified using the ITIS database (taxize package), these were merged (e.g., *Homalictus punctatus* was synonymised with *Lasioglossum punctatus*). Where the published phylogeny had species placements that appear very discrepant (i.e., the placement of *Ceratina japonica* and *Anthophora pillipes* outside of their otherwise monophyletic groups and placed with fairly distantly related species) and were noted as such by the authors of the tree (Hedtke *et al.*, 2013), these were considered missing species and their placement was estimated using pastis.

For twenty-eight bee clades with missing species (these were usually subfamilies or tribes which had greater than 85% bootstrap support; Table S2.1), a phylogenetic tree was developed using a birth-death model with zero extinction rate and exponential speciation rate using MrBayes, for at least 100,000,000 generations and four runs, with samples taken every 10000 generations. Tracer v1.4.1 was used to track effective sample sizes to assess convergence of parameter estimates. The standard deviation of split frequencies of the four independent runs were also assessed (with a value of less than 0.01 taken as evidence that the models were reaching convergence). From each of the converged runs, we then sub-sampled from the post-burn-in

posterior distribution of each bee clade to produce 1000 within-clade trees. A large clade of apid bees did not reach parameter convergence in any of the four independent runs, but measures of phylogenetic signal within the clade were not significantly different between runs (analysis of variance, $F_{df=3} = 1.27$, n.s.), so a random sample from all runs were taken. Random samples (without replacement) were then taken from the set of within-clade trees for each bee clade and were grafted onto the original phylogenetic tree. The original tree had first been rate smoothed, using PATHd8 (with the root age constrained to one) (Britton *et al.*, 2007), which is a computationally efficient methods for large phylogenetic trees, and incomplete clades were pruned. To graft the clades onto the original tree, the clade was first scaled to have a depth of 1; the edge lengths were then scaled by the age of the crown node of the clade.

Table S2.1: Details of bee clades (subsections of the tree, within each of which the placement of missing species was estimated using pastis (Thomas *et al.*, 2013) and MrBayes (Ronquist *et al.*, 2012)). Percentage bootstrap support for the node is shown for the genus tree from Hedtke *et al.* (2013) unless indicated otherwise. The number of generations run in MrBayes is also provided (some larger clades were run for longer in order to reach convergence).

Family	Clade	Bootstrap support	Generations (millions)
Apidae	Meloponini, Bombini, Apini	74	200
Apidae	Euglossini	100	100
Apidae	Centridini (Centris)	100	100
Apidae	Centridini (Epicharis)	100	100
Apidae	Allodapini and Ceratinini	100	100
Apidae	Manuelini	NA [1]	100
Apidae	Xylocopini	85 [2]	100

[1]No value available as only one species from this tribe is in the original tree
[2]Taken from species-level tree as only one genus is in this clade

Apidae	Epeolini, Brachynomadini, ammobatoidini, Biastini, Townsendiellini, Neolarrini, Nomadini, Hexepeoplini, Ammobatini, Melectini, Caenoprospidini, Ericrocidini, Rhathymini, Esepeolini, Osirini (Epeoloides), Protepeolini, Osirini (Osiris), Tetrapedini (Coelioxoides), Osirini (Parepeolus)	99	100
Apidae	Eucerini, Ancylini, Emphorini, Tapinotaspidini, Exomalopsini, Emphorini	100	100
Apidae	Anthophorini	100	100
Andrenidae	Andreninae	100	100
Andrenidae	Panurginae	99	100
Halictidae	Halictini	97	100
Halictidae	Sphecodini	100	100
Halictidae	Caenohalictini	99	100
Halictidae	Augochlorini	100	100
Halictidae	Nomiinae	100	100
Halictidae	Rophitinae	100	100
Halictidae	Nomiodinae	100	100
Melittidae	Melittidae	98	100
Colettidae	Hylaeinae, Scrapterinae, Euryglossinae, Xeromelissinae	97	200
Colettidae	Colletini	100	100
Colettidae	Neophashaeinae	100	100
Megachilidae	Megachilini	100	100
Megachilidae	Lithurgini	100	100

Megachilidae	Heriades	100	100
Megachilidae	Anthidini (anthidiellum & anthidini)	100	100
Megachilidae	Osmini	100	100

Appendix S3 Land-Use Intensity Maps

For maps of each land-use class (without land-use intensity), see Hoskins *et al.* (2016).

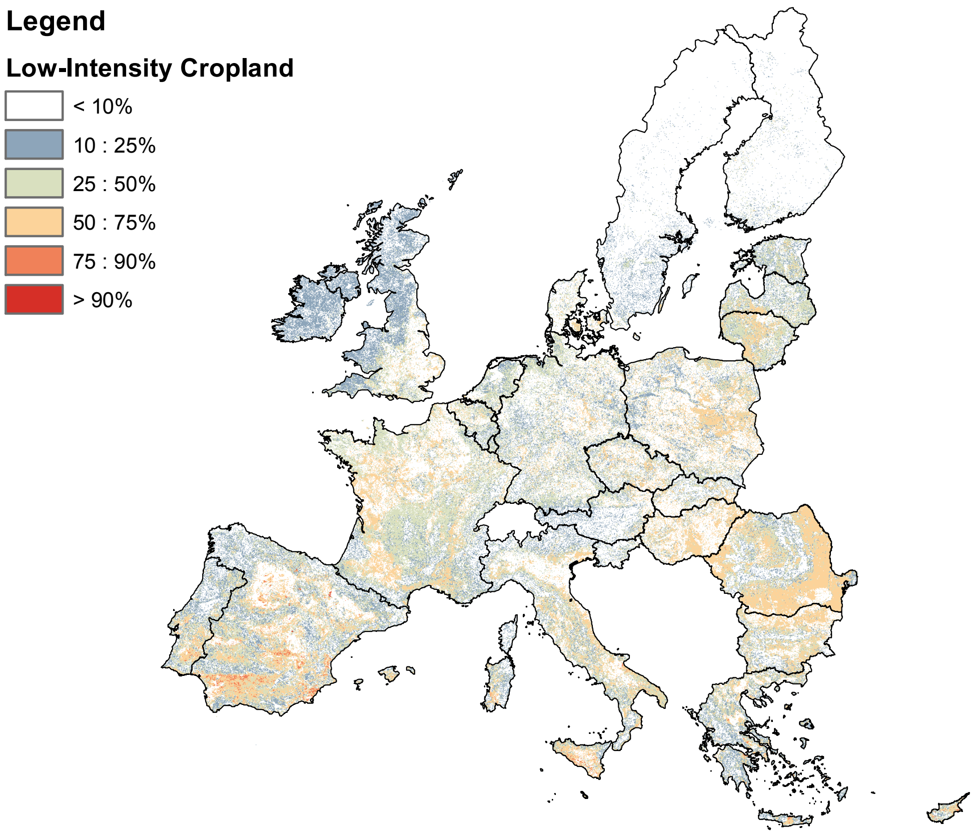

Figure S3.1: Percentage of each $1km^2$ grid cell that is low-intensity cropland.

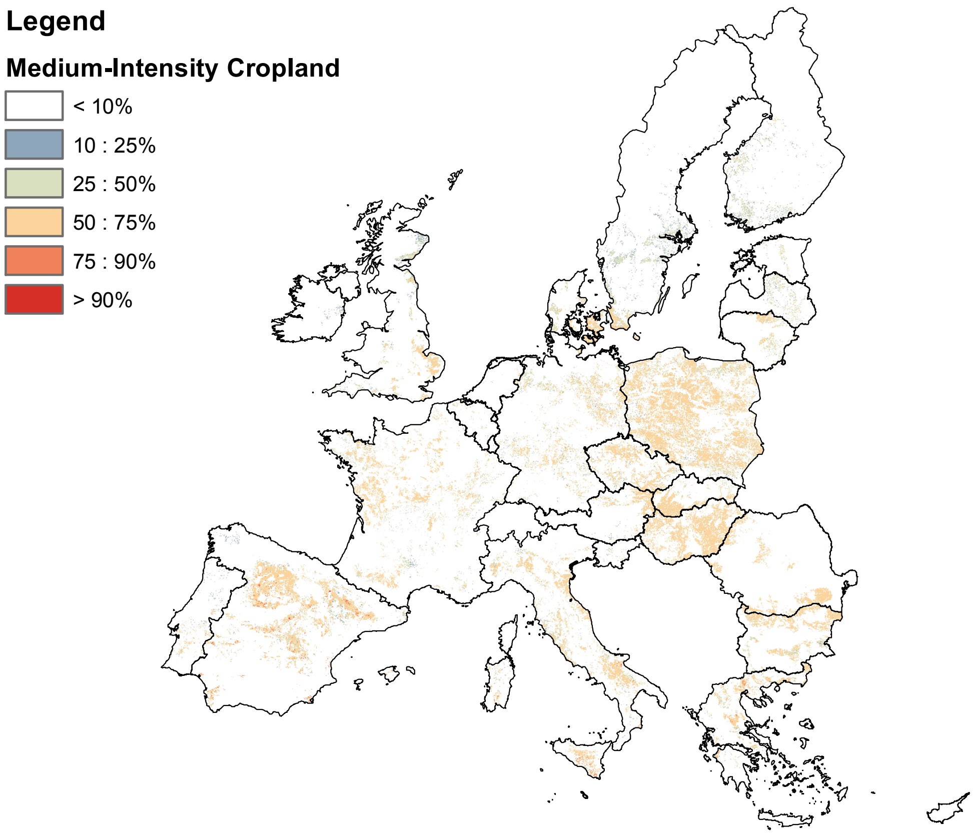

Figure S3.2: Percentage of each $1km^2$ grid cell that is medium-intensity cropland.

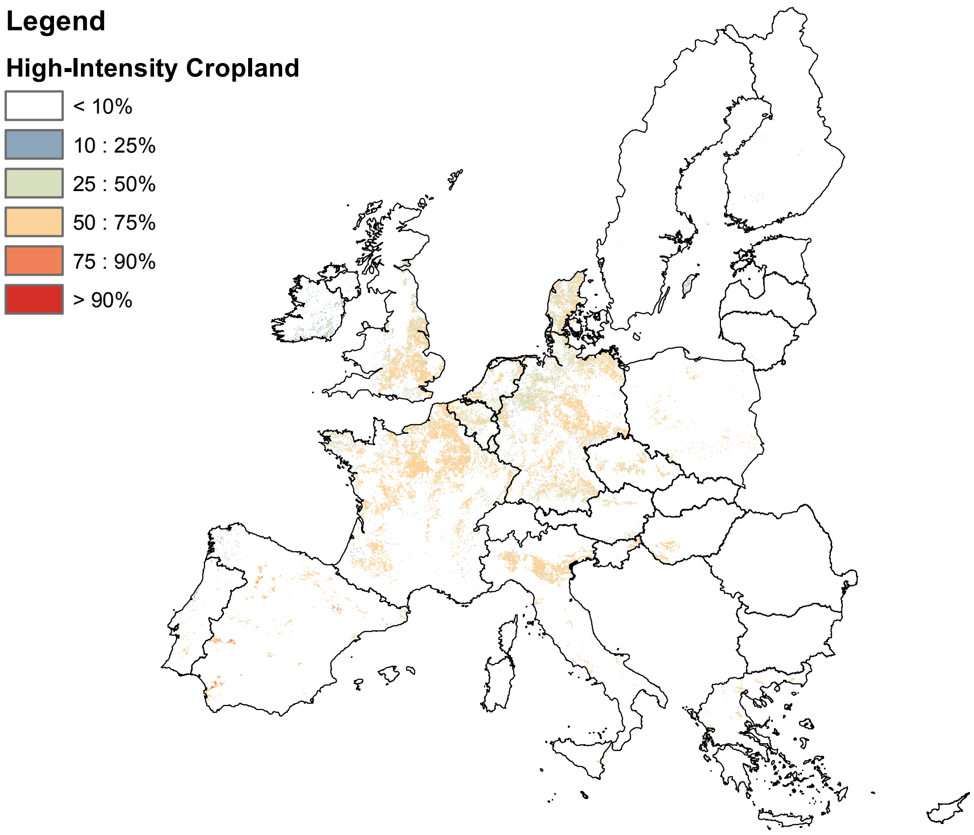

Figure S3.3: Percentage of each $1km^2$ grid cell that is high-intensity cropland.

Appendix S4 Supplementary Results

Appendix S4.1 Spatial Autocorrelation Results

Moran's I was used to assess spatial autocorrelation in model residuals (spdep package: Bivand & Piras, 2015; Bivand *et al.*, 2013). There was no significant evidence of spatial autocorrelation in the model residuals for species (Moran's I = -1.06, p = 0.86), functional (Moran's I = -0.94, p = 0.83) or phylogenetic diversity (Moran's I = -0.98, p = 0.83). Spatial autocorrelation was also assessed for the residuals of each study in turn. At most, two studies showed spatial autocorrelation in their residuals, but this was not more than expected by chance (one-sided χ^2 test: $\chi^2 = 5.0294e - 32, p = 0.5$).

Table S4.1: Coefficient estimates (calculated using treatment contrasts in R statistics) and bootstrapped statistics (standard error, bias, confidence intervals) for the model of log-transformed taxonomic diversity. Significance is assumed if the bootstrapped 95% confidence intervals do not cross zero (indicated by significance stars). The first level of the land-use and intensity factor (Natural/semi-natural vegetation) forms part of the intercept term and so does not explicitly appear in the coefficients table.

Coefficient	Original estimate	Standard error	bootstrapped Standard error	Bias	Lower 95% Confidence Interval	Upper 95% Confidence Interval	
Intercept	1.43	0.15	0.16	-0.01	1.14	1.72	*
Pasture	-0.39	0.15	0.16	0.01	-0.68	-0.10	*
Low-intensity cropland	-0.12	0.18	0.18	0.01	-0.47	0.25	
Medium-intensity cropland	-0.51	0.17	0.17	0.01	-0.85	-0.17	*
High-intensity cropland	-0.48	0.17	0.17	0.01	-0.82	-0.12	*
Urban	0.07	0.13	0.14	0.00	-0.20	0.33	

Table S4.2: Coefficient estimates (calculated using treatment contrasts in R statistics) and bootstrapped statistics (standard error, bias, confidence intervals) for the model of functional diversity. Significance is assumed if the bootstrapped 95% confidence intervals do not cross zero (indicated by significance stars). The first level of the land-use and intensity factor (Natural/semi-natural vegetation) forms part of the intercept term and so does not explicitly appear in the coefficients table. As log-transformed human population density (HPD) was scaled, the intercept is the mean logged HPD.

Coefficient	Original estimate	Standard error	bootstrapped Standard error	Bias	Lower 95% Confidence Interval	Upper 95% Confidence Interval	
Intercept	1.67	0.08	0.08	-0.00	1.52	1.84	*
Pasture	-0.15	0.08	0.08	-0.00	-0.32	-0.00	*
Low-intensity cropland	0.02	0.10	0.09	-0.00	-0.17	0.21	
Medium-intensity cropland	-0.18	0.09	0.09	0.00	-0.36	-0.02	*
High-intensity cropland	-0.17	0.09	0.09	0.00	-0.35	-0.01	*
Urban	-0.02	0.12	0.12	-0.00	-0.25	0.21	
Semi-natural vegetation: HPD	-0.84	1.18	1.14	0.01	-3.02	1.36	
Pasture: HPD	1.67	1.80	1.73	0.07	-1.75	5.26	
Low-intensity cropland: HPD	1.86	1.66	1.64	-0.02	-1.32	5.11	
Medium-intensity cropland: HPD	-3.40	1.62	1.65	-0.05	-6.71	-0.19	*
High-intensity cropland: HPD	1.68	1.51	1.44	-0.02	-1.16	4.75	
Urban: HPD	0.11	1.75	1.76	0.01	-3.40	3.58	

Table S4.3: Coefficient estimates (calculated using treatment contrasts in R statistics) and bootstrapped statistics (standard error, bias, confidence intervals) for the model of phylogenetic diversity. Significance is assumed if the bootstrapped 95% confidence intervals do not cross zero (indicated by significance stars). The first level of the land-use and intensity factor (Natural/semi-natural vegetation) forms part of the intercept term and so does not explicitly appear in the coefficients table. As log-transformed human population density (HPD) was scaled, the intercept is the mean logged HPD.

Coefficient	Original estimate	Standard error	bootstrapped Standard error	Bias	Lower 95% Confidence Interval	Upper 95% Confidence Interval	
Intercept	1.50	0.06	0.06	0.00	1.38	1.61	*
Pasture	-0.15	0.06	0.06	0.00	-0.27	-0.02	*
Low-intensity cropland	-0.03	0.07	0.07	0.00	-0.15	0.10	
Medium-intensity cropland	-0.16	0.06	0.06	0.00	-0.29	-0.03	*
High-intensity cropland	-0.15	0.06	0.06	0.00	-0.28	-0.03	*
Urban	-0.00	0.08	0.08	0.00	-0.16	0.15	
Semi-natural vegetation: HPD	-1.00	0.79	0.77	-0.02	-2.44	0.46	
Pasture: HPD	1.37	1.20	1.24	0.01	-1.03	3.75	
Low-intensity cropland: HPD	1.60	1.10	1.07	0.01	-0.38	3.73	
Medium-intensity cropland: HPD	-1.44	1.07	1.08	-0.00	-3.63	0.66	
High-intensity cropland: HPD	1.63	1.00	0.96	0.03	-0.23	3.50	
Urban: HPD	0.47	1.17	1.11	0.03	-1.69	2.70	

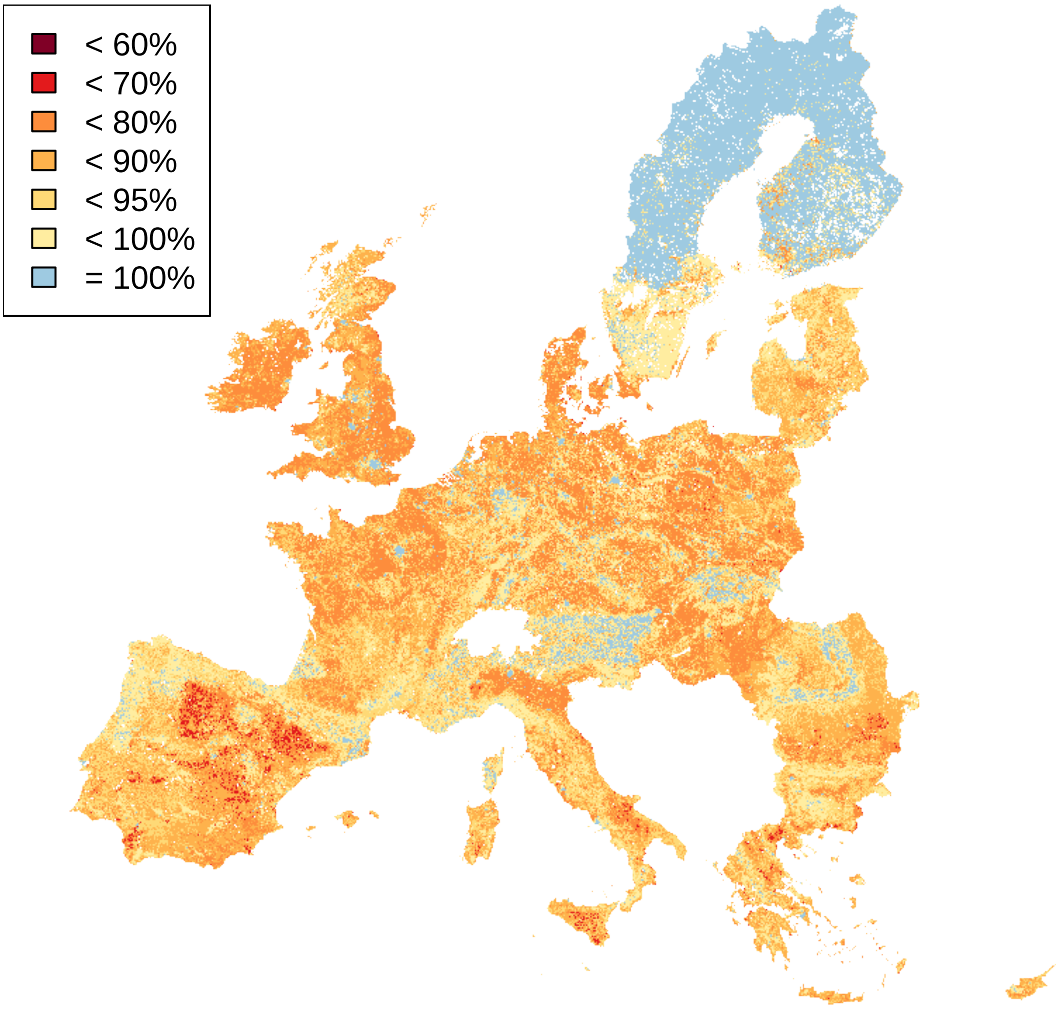

Figure S4.1: Percentage difference in species diversity in different land uses, relative to the natural/semi-natural baseline, for EU27 Countries at a $1km^2$ resolution.

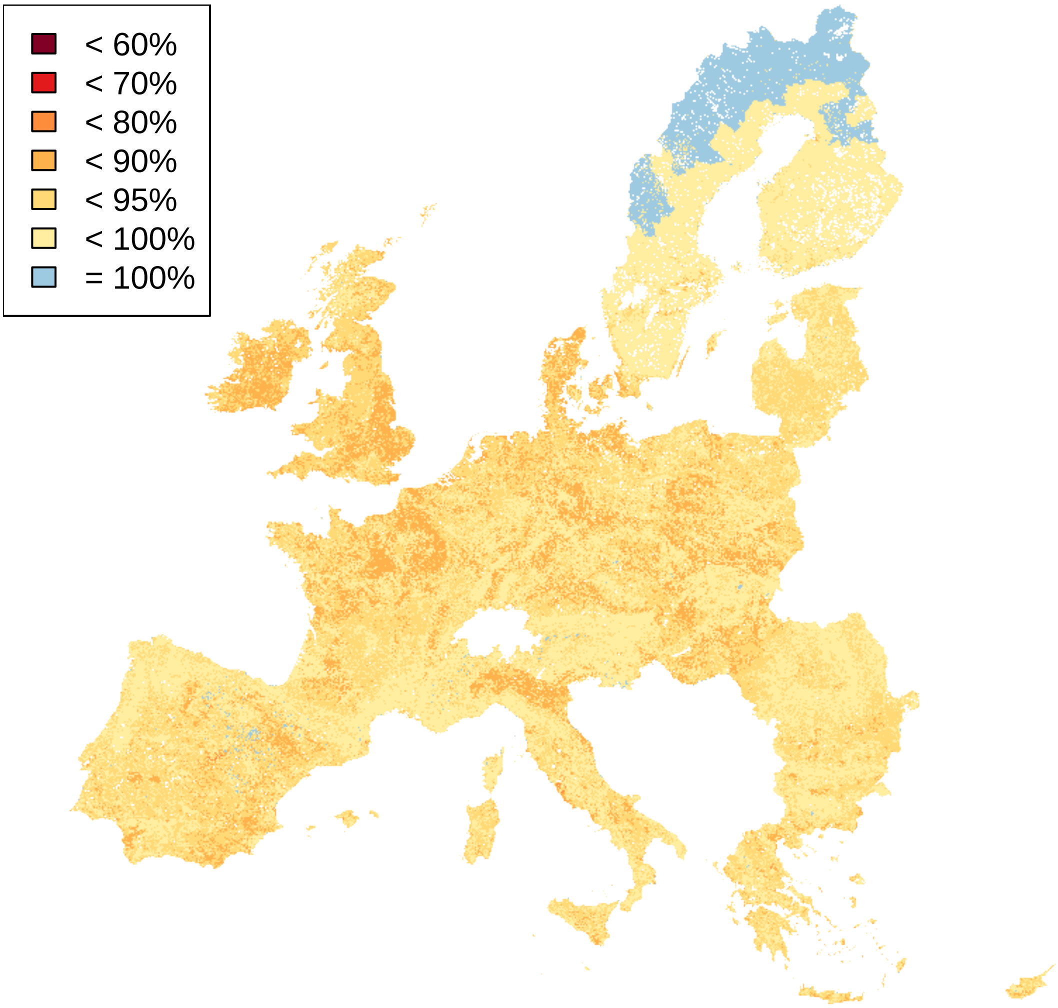

Figure S4.2: Percentage difference in functional diversity in different land uses, relative to the natural/semi-natural baseline, for EU27 Countries at a 1km^2 resolution.

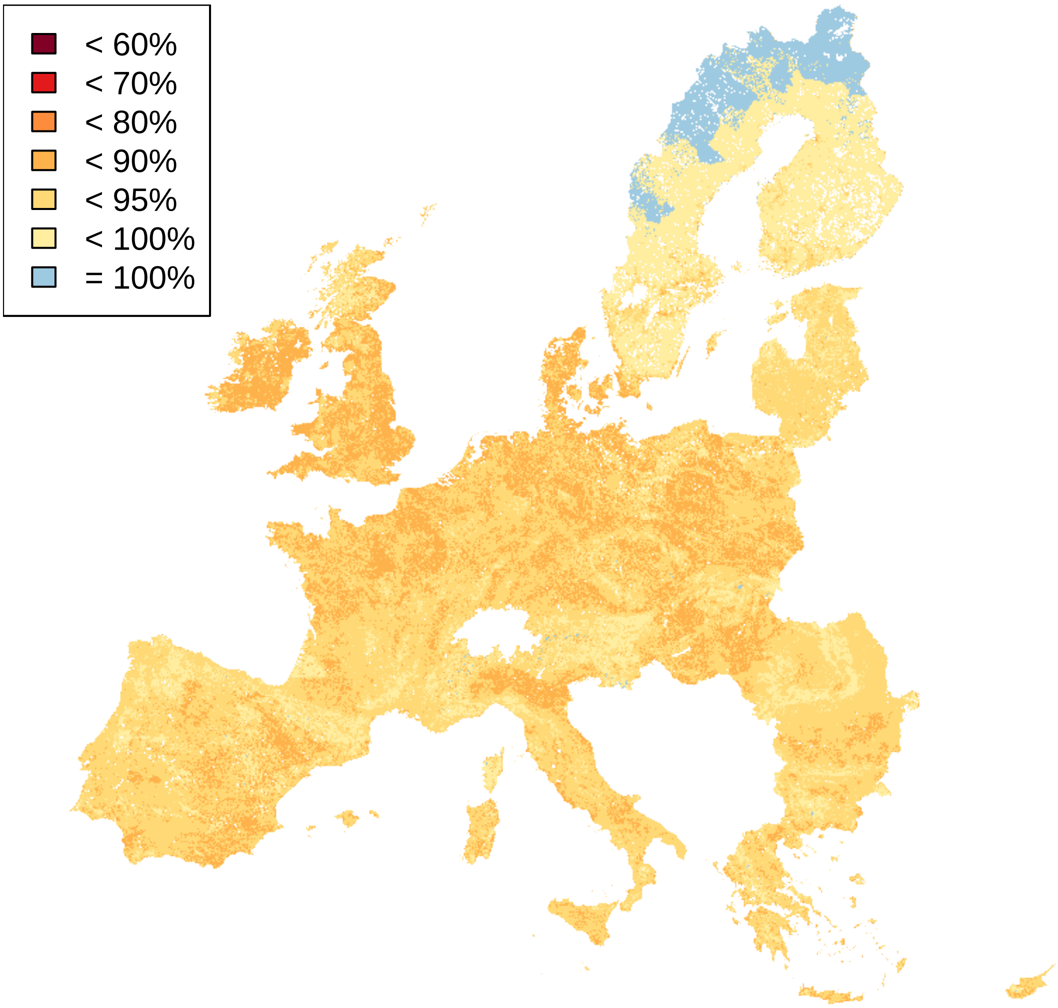

Figure S4.3: Percentage difference in phylogenetic diversity in different land uses, relative to the natural/semi-natural baseline, for EU27 Countries at a 1km^2 resolution.

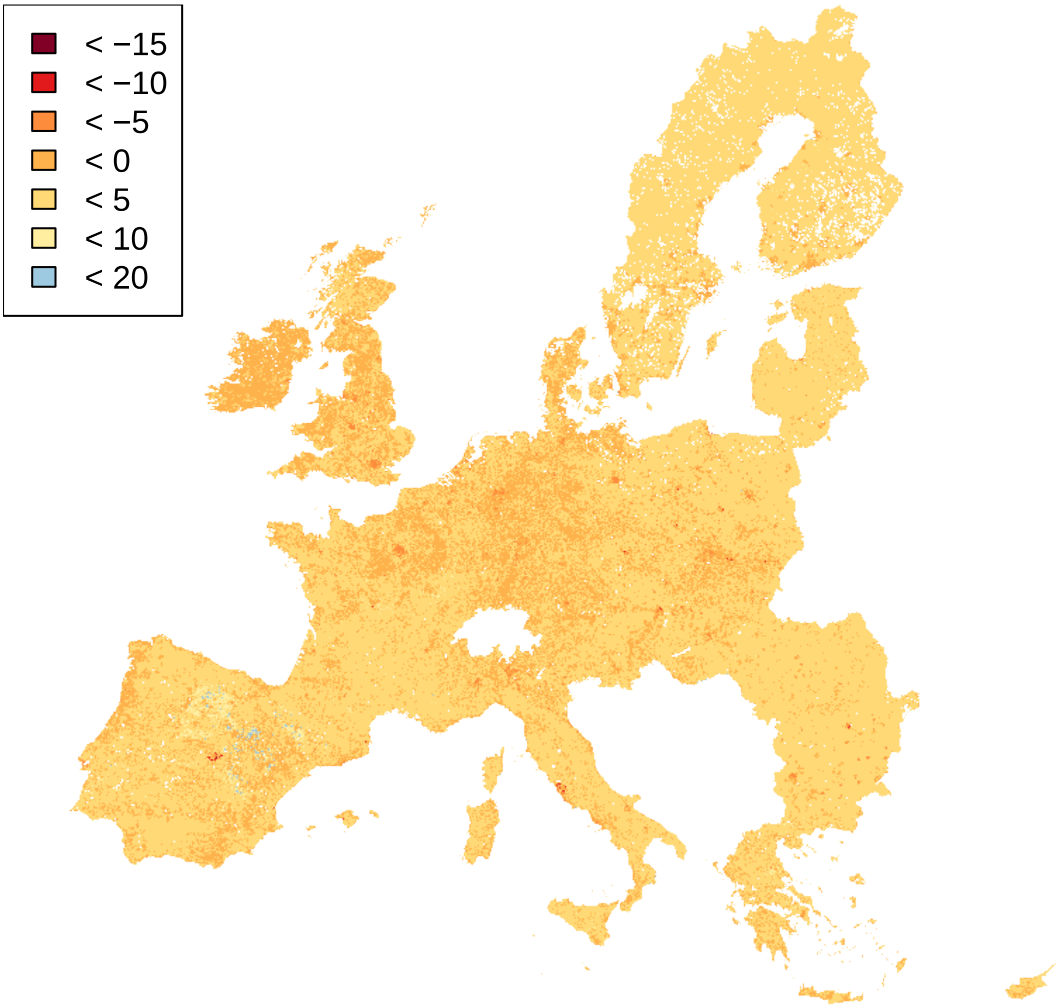

Figure S4.4: Residuals of a linear model of species diversity against functional diversity (i.e., a model of Fig. S4.1 against Fig. S4.2.

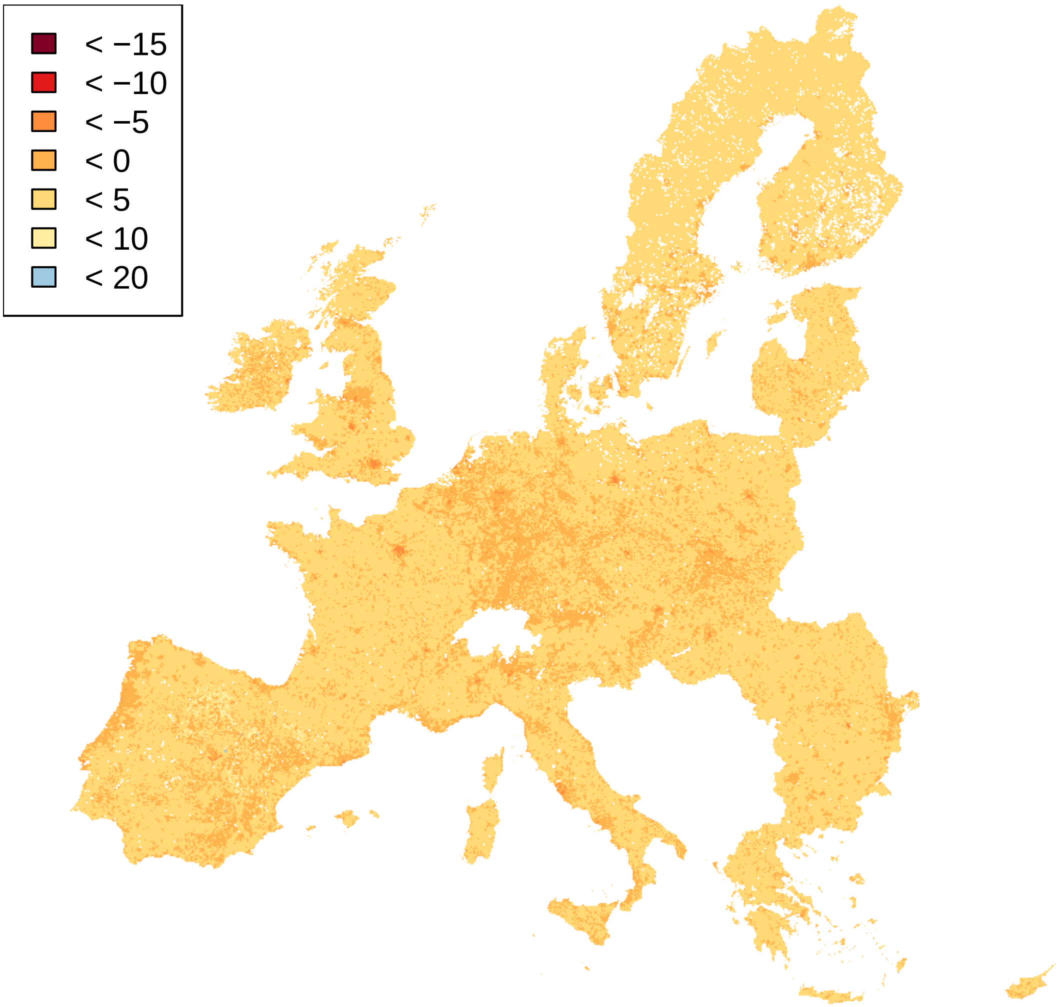

Figure S4.5: Residuals of a linear model of species diversity against phylogenetic diversity (i.e., a model of Fig. S4.1 against Fig. S4.3.

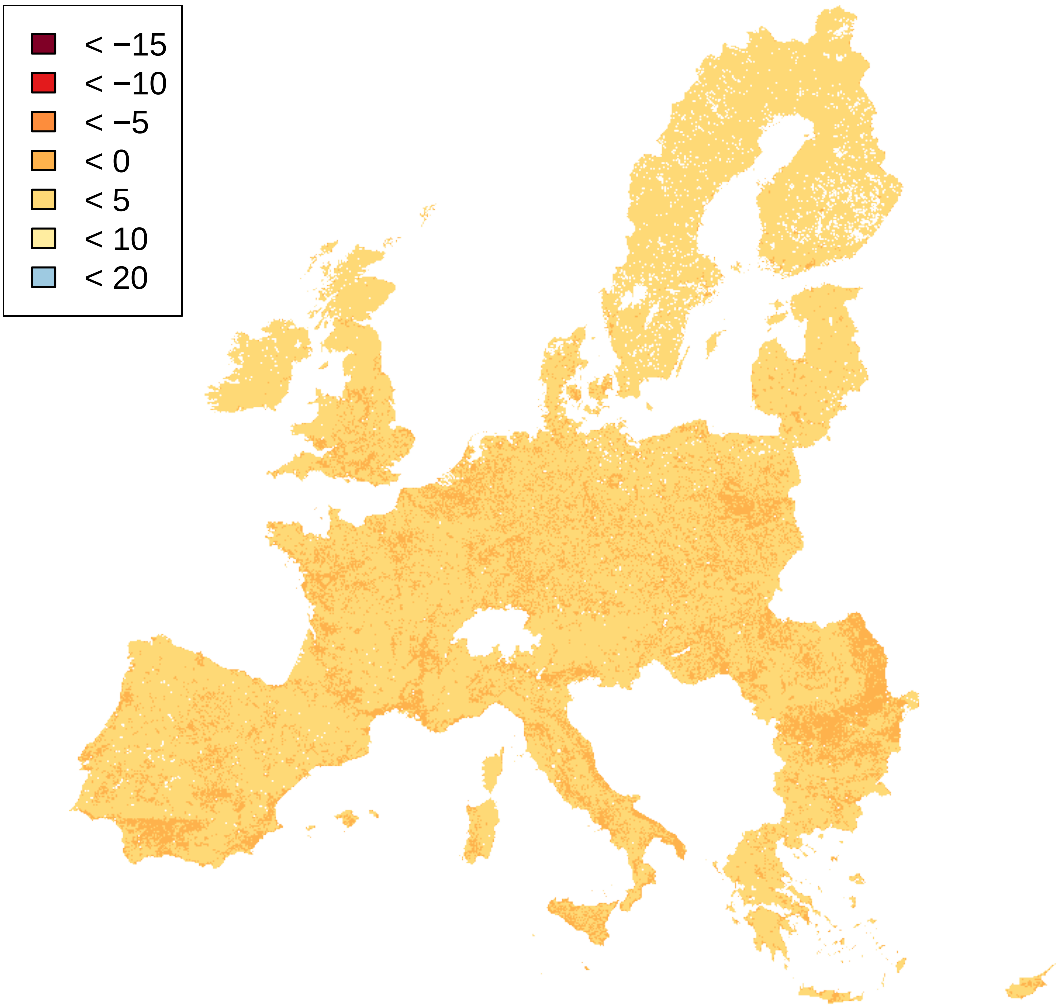

Figure S4.6: Residuals of a linear model of Fig. S4.4 against Fig. S4.5, that is, the difference between functional and phylogenetic diversity once correlations with species diversity are accounted for.

References

Albrecht, M., Schmid, B., Obrist, M.K., Schüpbach, B., Kleijn, D. & Duelli, P. (2010) Effects of ecological compensation meadows on arthropod diversity in adjacent intensively managed grassland, *Biological Conservation*, **143**:642–649.

Almeida, E. & Danforth, B.N. (2009) Phylogeny of colletid bees (Hymenoptera: Colletidae) inferred from four nuclear genes, *Molecular Phylogenetics and Evolution*, **50**:290–309.

Bates, A.J., Sadler, J.P., Fairbrass, A.J., Falk, S.J., Hale, J.D. & Matthews, T.J. (2011) Changing bee and hoverfly pollinator assemblages along an urban-rural gradient, *PLoS ONE*, **6**:e23459.

Billeter, R., Liira, J., Bailey, D., Bugter, R., Arens, P., Augenstein, I., Aviron, S., Baudry, J., Bukacek, R., Burel, F., Cerny, M., De Blust, G., De Cock, R., Diekötter, T., Dietz, H., Dirksen, J., Dormann, C., Durka, W., Frenzel, M., Hamersky, R., Hendrickx, F., Herzog, F., Klotz, S., Koolstra, B., Lausch, A., Le Coeur, D., Maelfait, J.P., Opdam, P., Roubalova, M., Schermann, A., Schermann, N., Schmidt, T., Schweiger, O., Smulders, M., Speelmans, M., Simova, P., Verboom, J., Van Wingerden, W., Zobel, M. & Edwards, P. (2007) Indicators for biodiversity in agricultural landscapes: a pan-European study, *Journal of Applied Ecology*, **45**:141–150.

Bivand, R. & Piras, G. (2015) Comparing Implementations of Estimation Methods for Spatial Econometrics, *Journal of Statistical Software*, **63**:1–36.

Bivand, R.S., Hauke, J. & Kossowski, T. (2013) Computing the Jacobian in Gaussian spatial autoregressive models: An illustrated comparison of available methods, *Geographical Analysis*, **45**:150–179.

Britton, T., Anderson, C.L., Jacquet, D., Lundqvist, S. & Bremer, K. (2007) Estimating divergence times in large phylogenetic trees, *Systematic biology*, **56**:741–752.

Danforth, B.N., Eardley, C., Packer, L., Walker, K., Pauly, A. & Randrianambinintsoa, F.J. (2008) Phylogeny of Halictidae with an emphasis on endemic African Halictinae, *Apidologie*, **39**:86–U42.

De Palma, A., Abrahamczyk, S., Aizen, M.A., Albrecht, M., Basset, Y., Bates, A., Blake, R.J., Boutin, C., Bugter, R., Connop, S., Cruz-López, L., Cunningham, S.A., Darvill, B., Diekötter, T., Dorn, S., Downing, N., Entling, M.H., Farwig, N., Felicioli, A., Fonte, S.J., Fowler, R., Franzén, M., Goulson, D., Grass, I., Hanley, M.E., Hendrix, S.D., Herrmann, F., Herzog, F., Holzschuh, A., Jauker, B., Kessler, M., Knight, M.E., Kruess, A., Lavelle, P., Le Féon, V., Lentini, P., Malone, L.A., Marshall, J., Pachón, E.M., McFrederick, Q.S., Morales, C.L., Mudri-Stojnic, S., Nates-Parra, G., Nilsson, S.G., Öckinger, E., Osgathorpe, L., Parra-H, A., Peres, C.A., Persson, A.S., Petanidou, T., Poveda, K., Power, E.F., Quaranta, M., Quintero, C., Rader, R., Richards, M.H., Roulston, T., Rousseau, L., Sadler, J.P., Samnegård, U., Schellhorn, N.A., Schüepp, C., Schweiger, O., Smith-Pardo, A.H., Steffan-Dewenter, I., Stout, J.C., Tonietto, R.K., Tscharntke, T., Tylianakis, J.M., Verboven, H.A.F., Vergara, C.H., Verhulst, J., Westphal, C., Yoon, H.J. & Purvis, A. (2016) Predicting bee community responses to land-use changes: Effects of geographic and taxonomic biases, *Scientific Reports*, **6**:31153.

Diekötter, T., Billeter, R. & Crist, T.O. (2008) Effects of landscape connectivity on the spatial distribution of insect diversity in agricultural mosaic landscapes, *Basic and Applied Ecology*, **9**:298–307.

Fowler, R.E. (2014) *An investigation into bee assemblage change along an urban-rural gradient*, Ph.D. thesis.

Franzén, M. & Nilsson, S.G. (2008) How can we preserve and restore species richness of pollinating insects on agricultural land?, *Ecography*, **31**:698–708.

Hanley, M. (2011) Unpublished data of bee diversity in UK croplands and urban habitats.

Hedtke, S.M., Patiny, S. & Danforth, B.N. (2013) The bee tree of life: a supermatrix approach to apoid phylogeny and biogeography, *BMC Evolutionary Biology*, **13**:138.

Holzschuh, A., Dormann, C.F., Tscharntke, T., Steffan-Dewenter, I. & B, P.R.S. (2011) Expansion of mass-flowering crops leads to transient pollinator dilution and reduced wild plant pollination, *Proceedings of the Royal Society B: Biological Sciences*, **278**:3444–3451.

Hoskins, A.J., Bush, A., Gilmore, J., Harwood, T., Hudson, L.N., Ware, C., Williams, K.J. & Ferrier, S. (2016) Downscaling land-use data to provide global 30??? estimates of five land-use classes, *Ecology and Evolution*:n/a–n/a.

Hudson, L.N., Newbold, T., Contu, S., Hill, S.L.L., Lysenko, I., De Palma, A., Phillips, H.R.P., Senior, R.A., Bennett, D.J., Booth, H., Choimes, A., Correia, D.L.P., Day, J., Echeverría-Londoño, S., Garon, M., Harrison, M.L.K., Ingram, D.J., Jung, M., Kemp, V., Kirkpatrick, L., Martin, C.D., Pan, Y., White, H.J., Aben, J., Abrahamczyk, S., Adum, G.B., Aguilar-Barquero, V., Aizen, M.A., Ancrenaz, M., Arbeláez-Cortés, E., Armbrecht, I., Azhar, B., Azpiroz, A.B., Baeten, L., Báldi, A., Banks, J.E., Barlow, J., Batáry, P., Bates, A.J., Bayne, E.M., Beja, P., Berg, Å., Berry, N.J., Bicknell, J.E., Bihn, J.H., Böhning-Gaese, K., Boekhout, T., Boutin, C., Bouyer, J., Brearley, F.Q., Brito, I., Brunet, J., Buczkowski, G., Buscardo, E., Cabra-García, J., Calviño-Cancela, M., Cameron, S.A., Cancello, E.M., Carrijo, T.F., Carvalho, A.L., Castro, H., Castro-Luna, A.A., Cerda, R., Cerezo, A., Chauvat, M., Clarke, F.M., Cleary, D.F.R., Connop, S.P., D'Aniello, B., da Silva, P.G., Darvill, B., Dauber, J., Dejean, A., Diekötter, T., Dominguez-Haydar, Y., Dormann, C.F., Dumont, B., Dures, S.G., Dynesius, M., Edenius, L., Elek, Z., Entling, M.H., Farwig, N., Fayle, T.M., Felicioli, A., Felton, A.M., Ficetola, G.F., Filgueiras, B.K.C., Fonte, S.J., Fraser, L.H., Fukuda, D., Furlani, D., Ganzhorn, J.U., Garden, J.G., Gheler-Costa, C., Giordani, P., Giordano, S., Gottschalk, M.S., Goulson, D., Gove, A.D., Grogan, J., Hanley, M.E., Hanson, T., Hashim, N.R., Hawes, J.E., Hébert, C., Helden, A.J., Henden, J.A., Hernández, L., Herzog, F., Higuera-Diaz, D., Hilje, B., Horgan, F.G., Horváth, R., Hylander, K., Isaacs-Cubides, P., Ishitani, M., Jacobs, C.T., Jaramillo, V.J., Jauker, B., Jonsell, M., Jung, T.S., Kapoor, V., Kati, V., Katovai, E., Kessler, M., Knop, E., Kolb, A., Korösi, Á., Lachat, T., Lantschner, V., Le Féon, V., LeBuhn, G., Légaré, J.P., Letcher, S.G., Littlewood, N.A., López-Quintero, C.A., Louhaichi, M., Lövei, G.L., Lucas-Borja, M.E., Luja, V.H., Maeto, K., Magura, T., Mallari, N.A., Marin-Spiotta, E., Marshall, E.J.P., Martínez, E., Mayfield, M.M., Mikusinski, G., Milder, J.C., Miller, J.R., Morales, C.L., Muchane, M.N., Muchane, M., Naidoo, R., Nakamura, A., Naoe, S., Nates-Parra, G., Navarrete Gutierrez, D.A., Neuschulz, E.L., Noreika, N., Norfolk, O., Noriega, J.A., Nöske, N.M., O'Dea, N., Oduro, W., Ofori-Boateng, C., Oke, C.O., Osgathorpe, L.M.,

Paritsis, J., Parra-H, A., Pelegrin, N., Peres, C.A., Persson, A.S., Petanidou, T., Phalan, B., Philips, T.K., Poveda, K., Power, E.F., Presley, S.J., Proença, V., Quaranta, M., Quintero, C., Redpath-Downing, N.A., Reid, J.L., Reis, Y.T., Ribeiro, D.B., Richardson, B.A., Richardson, M.J., Robles, C.A., Römbke, J., Romero-Duque, L.P., Rosselli, L., Rossiter, S.J., Roulston, T.H., Rousseau, L., Sadler, J.P., Sáfián, S., Saldaña-Vázquez, R.A., Samnegård, U., Schüepp, C., Schweiger, O., Sedlock, J.L., Shahabuddin, G., Sheil, D., Silva, F.A.B., Slade, E.M., Smith-Pardo, A.H., Sodhi, N.S., Somarriba, E.J., Sosa, R.A., Stout, J.C., Struebig, M.J., Sung, Y.H., Threlfall, C.G., Tonietto, R., Tóthmérész, B., Tscharntke, T., Turner, E.C., Tylianakis, J.M., Vanbergen, A.J., Vassilev, K., Verboven, H.A.F., Vergara, C.H., Vergara, P.M., Verhulst, J., Walker, T.R., Wang, Y., Watling, J.I., Wells, K., Williams, C.D., Willig, M.R., Woinarski, J.C.Z., Wolf, J.H.D., Woodcock, B.A., Yu, D.W., Zaitsev, A.S., Collen, B., Ewers, R.M., Mace, G.M., Purves, D.W., Scharlemann, J.P.W. & Purvis, A. (2014) The PREDICTS database: a global database of how local terrestrial biodiversity responds to human impacts, *Ecology and Evolution*, **4**:4701–4735.

Hudson, L.N., Newbold, T., Contu, S., Hill, S.L.L., Lysenko, I., De Palma, A., Phillips, H.R.P., Alhusseini, T.I., Bedford, F.E., Bennett, D.J., Booth, H., Burton, V.J.,... & Purvis, A. (2014) The database of the PREDICTS (Projecting Responses of Ecological Diversity in Changing Terrestrial Systems) project, *Ecology and Evolution*, **7**:145–188.

Jauker, B., Krauss, J., Jauker, F. & Steffan-Dewenter, I. (2013) Linking life history traits to pollinator loss in fragmented calcareous grasslands, *Landscape Ecology*, **28**:107–120.

Kohler, F., Verhulst, J., Van Klink, R. & Kleijn, D. (2007) At what spatial scale do high-quality habitats enhance the diversity of forbs and pollinators in intensively farmed landscapes?, *Journal of Applied Ecology*, **45**:753–762.

Kruess, A. & Tscharntke, T. (2002) Grazing Intensity and the Diversity of Grasshoppers, Butterflies, and Trap-Nesting Bees and Wasps, *Conservation Biology*, **16**:1570–1580.

Le Féon, V., Schermann-Legionnet, A., Delettre, Y., Aviron, S., Billeter, R., Bugter, R., Hendrickx, F. & Burel, F. (2010) Intensification of agriculture, landscape composition and wild

bee communities: A large scale study in four European countries, *Agriculture Ecosystems & Environment*, **137**:143–150.

Marshall, E., West, T. & Kleijn, D. (2006) Impacts of an agri-environment field margin prescription on the flora and fauna of arable farmland in different landscapes, *Agriculture, Ecosystems & Environment*, **113**:36–44.

Meyer, B., Gaebele, V. & Steffan-Dewenter, I.D. (2007) Patch Size and Landscape Effects on Pollinators and Seed Set of the Horseshoe Vetch, Hippocrepis comosa, in an Agricultural Landscape of Central Europe, *Entomologia Generalis*, **30**:173–185.

Meyer, B., Jauker, F. & Steffan-Dewenter, I. (2009) Contrasting resource-dependent responses of hoverfly richness and density to landscape structure, *Basic and Applied Ecology*, **10**:178–186.

Michener, C.D. (2000) *The Bees of the World*, The John Hopkins University Press, London, 913 pp.

Mudri-Stojnic, S., Andric, A., Józan, Z. & Vujic, A. (2012) Pollinator diversity (Hymenoptera and Diptera) in semi-natural habitats in Serbia during summer, *Archives of Biological Sciences*, **64**:777–786.

Power, E.F. & Stout, J.C. (2011) Organic dairy farming: impacts on insect-flower interaction networks and pollination, *Journal of Applied Ecology*, **48**:561–569.

Quaranta, M., Ambroselli, S., Barro, P., Bella, S., Carini, A., Celli, G., Cogoi, P., Comba, L., Comoli, R., Felicioli, A., Floris, I., Intoppa, F., Longo, S., Maini, S., Manino, A., Mazzeo, G., Medrzycki, P., Nardi, E., Niccolini, L., Palmieri, N., Patetta, A., Piatti, C., Piazza, M., Pinzauti, M., Porporato, M., Porrini, C., Ricciardelli d'Albore, G., Romagnoli, F., Ruiu, L., Satta, A. & Zandigiacomo, P. (2004) Wild bees in agroecosystems and semi-natural landscapes. 1997-2000 collection period in Italy, *Bulletin of Insectology*, **57**:11–61.

Ronquist, F., Teslenko, M., van der Mark, P., Ayres, D.L., Darling, A., Hohna, S., Larget, B., Liu, L., Suchard, M.A. & Huelsenbeck, J.P. (2012) MrBayes 3.2: Efficient Bayesian Phylogenetic Inference and Model Choice Across a Large Model Space, *Systematic Biology*, **61**:539–542.

Samnegård, U., Persson, A.S. & Smith, H.G. (2011) Gardens benefit bees and enhance pollination in intensively managed farmland, *Biological Conservation*, **144**:2602–2606.

Schüepp, C., Herrmann, J.D., Herzog, F. & Schmidt-Entling, M.H. (2011) Differential effects of habitat isolation and landscape composition on wasps, bees, and their enemies, *Oecologia*, **165**:713–721.

Thomas, G.H., Hartmann, K., Jetz, W., Joy, J.B., Mimoto, A. & Mooers, A.O. (2013) PASTIS: an R package to facilitate phylogenetic assembly with soft taxonomic inferences, *Methods in Ecology and Evolution*, **4**:1011–1017.

Weiner, C.N., Werner, M., Linsenmair, K.E. & Blüthgen, N. (2011) Land use intensity in grasslands: Changes in biodiversity, species composition and specialisation in flower visitor networks, *Basic and Applied Ecology*, **12**:292–299.
